# Template-assisted synthesis of pH-responsive hollow mesoporous silica nanocarriers: the role of engineered pores and surface characteristics

**DOI:** 10.1007/s10856-025-06995-z

**Published:** 2026-01-20

**Authors:** Sahar Gooneh-Farahani, Mohammad Imani, Morteza Daliri Joupari, Abdolreza Simchi

**Affiliations:** 1 Center for Nanoscience and Nanotechnology, Institute for Convergence Science & Technology, Sharif University of Technology, 14588 89694 Tehran, Iran; 2 Animal and Marine Biotechnology Dept., National Institute of Genetic Engineering and Biotechnology, 14977 16316 Tehran, Iran; 3 Department of Materials Science and Engineering, Sharif University of Technology, 14588 89694 Tehran, Iran; 4 Fraunhofer Institute for Manufacturing Technology and Advanced Materials (IFAM), 28359 Bremen, Germany

**Keywords:** Smart amorphous silica, Hollow nanoparticles, Surface modification, Pore engineering, Stimuli-responsive nanocarriers

## Abstract

Hollow silica nanoparticles (HSNPs), characterized by a hollow interior enclosed within a solid mesoporous silica shell, offer several advantages, including low density, high surface area, excellent adsorption capacity, and biocompatibility, making them highly attractive for diverse applications in fields such as food, construction, electronics, imaging, and nanomedicine. To investigate the largely unexplored role of the hollow interior and surface functionality in the design of smart nanocarriers, we propose a facile, green-chemistry-based approach for the synthesis of HSNPs, utilizing polystyrene nanoparticles (64 ± 11 nm in diameter) as sacrificial templates. An ultrathin mesoporous silica shell, 10–12 nm in thickness, is conformally deposited through the controlled hydrolysis of a Si precursor, yielding a nanocarrier system that enables the high adsorption of macromolecules with a pH-sensitive desorption profile. Comprehensive analytical techniques reveal that the method of template removal significantly influences both the interior and exterior pore structures. Notably, calcination produces HSNPs with a higher specific surface area ( > 195 m² g⁻¹), a larger average pore diameter ( ~ 20 nm), and an ink-bottle-like mesoporous structure. It is shown that these structural differences, combined with tailored surface functionalities, critically modulate the triggering response of the nanocarrier. To demonstrate functionality, doxorubicin hydrochloride (DOX) was employed as a model drug. A pH-responsive desorption behavior, releasing the biomacromolecule four times faster at pH=4.5 than at pH=7.4, is presented. This finding underscores the impact of surface chemistry and pore architecture on the adsorption and desorption kinetics of macromolecules. The results of this study pave the way for the rational design of stimuli-responsive ceramic nanocarriers with enhanced adsorption efficiency and precise, controlled desorption capabilities.

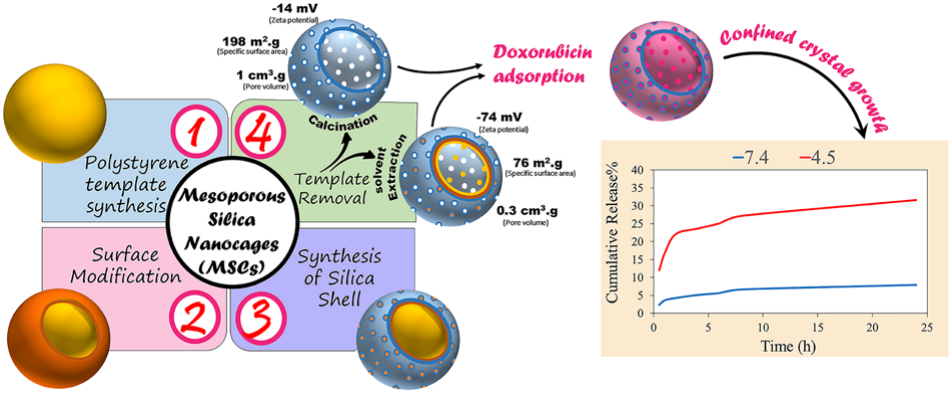

## Introduction

Amorphous silica nanoparticles are among the most widely used inorganic materials due to their exceptional properties, including chemical stability, mechanical durability, porosity, high surface area, biocompatibility, and non-toxicity [1–3]. A notable advancement in this field is the development of hollow silica nanoparticles (HSNPs), which, in addition to micro- and/or mesoporous structures, contain an internal cavity that provides further functional advantages [4]. For example, HSNPs possess an increased specific surface area, resulting in enhanced adsorption capacity due to more accessible surface sites [5]. The internal cavity also acts as an air reservoir with low thermal conductivity, making HSNPs well-suited for thermal insulation applications [6, 7]. Furthermore, their low refractive index enhances their performance in transparent coating applications [8, 9]. In biomedical and nanomedicine contexts, particularly drug delivery, the reduced concentration of HSNPs required for efficacy helps minimize the risk of nanoparticle-induced toxicity [10–12].

One of the most compelling features of hollow silica nanoparticles (HSNPs) is their exceptional structural tunability, which makes them highly versatile for a broad spectrum of applications. To fully exploit this potential, key architectural parameters, such as pore size and volume, shell thickness, and surface chemistry, must be precisely engineered, as these factors critically influence the nanoparticles' functional performance. By carefully adjusting different synthesis parameters, these structural features can be effectively tuned to achieve the desired porosity and surface characteristics. For example, in the study by Rizzi et al. on the modulation of silica nanoparticle size, it was shown that variations in base concentration, reaction time, temperature, and ageing conditions significantly affect both particle size and pore characteristics [13]. He et al. also demonstrated that the surface area and pore size of mesoporous silica nanoparticles can be effectively tuned by adjusting the synthesis parameters, such as the molar ratio of surfactant to silica precursor and the ethanol-to-water volume ratio. They showed that by increasing both the surfactant-to-precursor molar ratio and the ethanol-to-water ratio, the pore size could be increased from 2.4 to 6.5 nm [14]. Sun et al. investigated the effect of synthesis parameters on the particle size and mesopore structure of mesoporous silica nanoparticles. They found that by adjusting the amounts of 1st ethanol added to the reaction mixture, both the particle size and pore diameter could be controlled. The synthesized nanoparticles exhibited particle sizes ranging from 98 to 278 nm, with tunable mesopore sizes between 7.0 and 21.0 nm [15].

The pore characteristics, such as the size and shape of porous materials, including silica nanoparticles, affect their performance. For example, Pérez-Moreno et al. investigated the effect of pore size in mesoporous silica nanoparticles on their performance as carriers for the drug rapamycin. They demonstrated that nanoparticles with smaller pores exhibited higher drug-loading capacities, while those with larger pores showed faster drug release kinetics [16]. Jia et al. studied the drug release behavior of paclitaxel from mesoporous silica nanoparticles with three different pore sizes. They found that nanoparticles with larger pores exhibited a faster drug release rate compared to those with smaller pores [17]. According to the IUPAC classification, H1-type hysteresis corresponds to uniform cylindrical pores with open networks; H2-type is associated with pore-blocking effects and is related to ink-bottle–shaped pores. H3-type hysteresis is often found in non-rigid aggregates of plate-like particles that give rise to slit-shaped pores, while H4-type is typically observed in narrow slit-like micropores [18]. Understanding these hysteresis behaviors is essential for rational pore engineering, as they link the microscopic structure of silica nanoparticles to their macroscopic functional properties. For example, when used as nanocarriers, attributes like loading capacity, release kinetics, and responsiveness to external stimuli are strongly governed by the internal pore structure and surface properties. However, despite these customizable features, relatively few studies have systematically investigated how deliberate control over pore architecture and surface characteristics can be harnessed to design intelligent, stimuli-responsive nanosystems. A deeper understanding of these intrinsic design parameters could pave the way for next-generation nanocarriers with tailored environmental responsiveness and enhanced therapeutic efficacy.

Various strategies have been explored to develop smart silica nanoparticles, though success has generally been limited to moderate. For example, the functionalization of HSNPs with amine groups has been shown to enhance loading capacity by approximately 20% and encapsulation efficiency by up to 97% compared to non-functionalized counterparts [19]. However, such nanocarriers often exhibit weak pH-responsive release profiles. Enhanced performance has been reported through functionalization with hyaluronic acid (HA), achieved by anchoring dopamine to HSNP-B(OH)₂ *via* boronate ester bonds [20]. Studies using model biomacromolecules such as doxorubicin (DOX) and indocyanine green (ICG) have demonstrated increased loading efficiency and pH-triggered release at acidic conditions (pH 5) compared to physiological pH (7.4) over 72 hours. Strong pH-responsive behavior has also been observed in polydopamine-modified HSNPs [21], with DOX release rates increasing by 4–5 fold when the pH is reduced from 7.4 to 5. Similarly, polystyrene sulfonate has been employed to create pH-sensitive nanocarriers *via* protonation and swelling of the cationized polymer [22]; however, these systems showed a significant burst release, 22.3% at pH 5.5 versus 9.9% at pH 7.4 over 43 hours. Another approach using biomimetic membranes containing pH-sensitive lipids achieved a fourfold increase in DOX release at pH 5.5 ( ~ 20%) [23].

This brief literature survey highlights that, despite the advantages conferred by high surface area and improved adsorption capacity, the pH-responsive release behavior of HSNPs remains suboptimal and underdeveloped. In particular, current methods relying on surface chemical modifications often involve complex synthesis procedures, raise potential toxicity concerns, and suffer from limited long-term stability. Recent advances in nanocarrier design include bio-inspired and hybrid systems, which aim to improve targeting efficiency, biocompatibility, and controlled release. A bio-inspired strategy involves coating nanocarriers with biological membranes, which can enhance biocompatibility and help ensure the safe and effective performance of the designed carriers [24]. For example, the use of stem cells as carriers for delivering various types of drugs has been investigated [25–28]. In addition, the use of cell membranes to coat synthetic nanocarriers has been explored as a strategy to overcome challenges such as immunogenicity and toxicity [29–32]. However, these approaches are still in the early stages of research, and challenges remain regarding the standardization and improvement of the separation and purification, storage stability, and the complexity of their production [33]. The development of hybrid nanocarriers can further enable targeted delivery and more effective therapy by tuning their physicochemical properties [34]. For instance, several hybrid nanocarrier systems, such as polymer-coated mesoporous silica nanoparticles [35, 36], lipid-polymer hybrids [37, 38], and metal-organic framework (MOF)-based composites [39, 40], have been developed for controlled and pH-responsive drug release. While these systems demonstrate improved functionality, they often rely on complex fabrication processes, exhibit limited reproducibility. To the authors’ knowledge, the intrinsic potential of tuning the nanoparticle structure itself, specifically the pore network and shell architecture, to achieve pH-responsive behavior without external surface modification has been largely overlooked and warrants further investigation. This research focuses on the pore structure and surface properties of HSNPs using facile chemical routes, without the need for additional chemical agents. Unlike conventional strategies that depend on surface functionalization, the novelty of our approach lies in inducing pH-sensitive behavior purely through structural design. Specifically, the rational engineering of the mesoporous shell and intrinsic surface characteristics of HSNPs enables controlled, pH-dependent drug release without requiring further chemical modification.

**Fig. 1:**
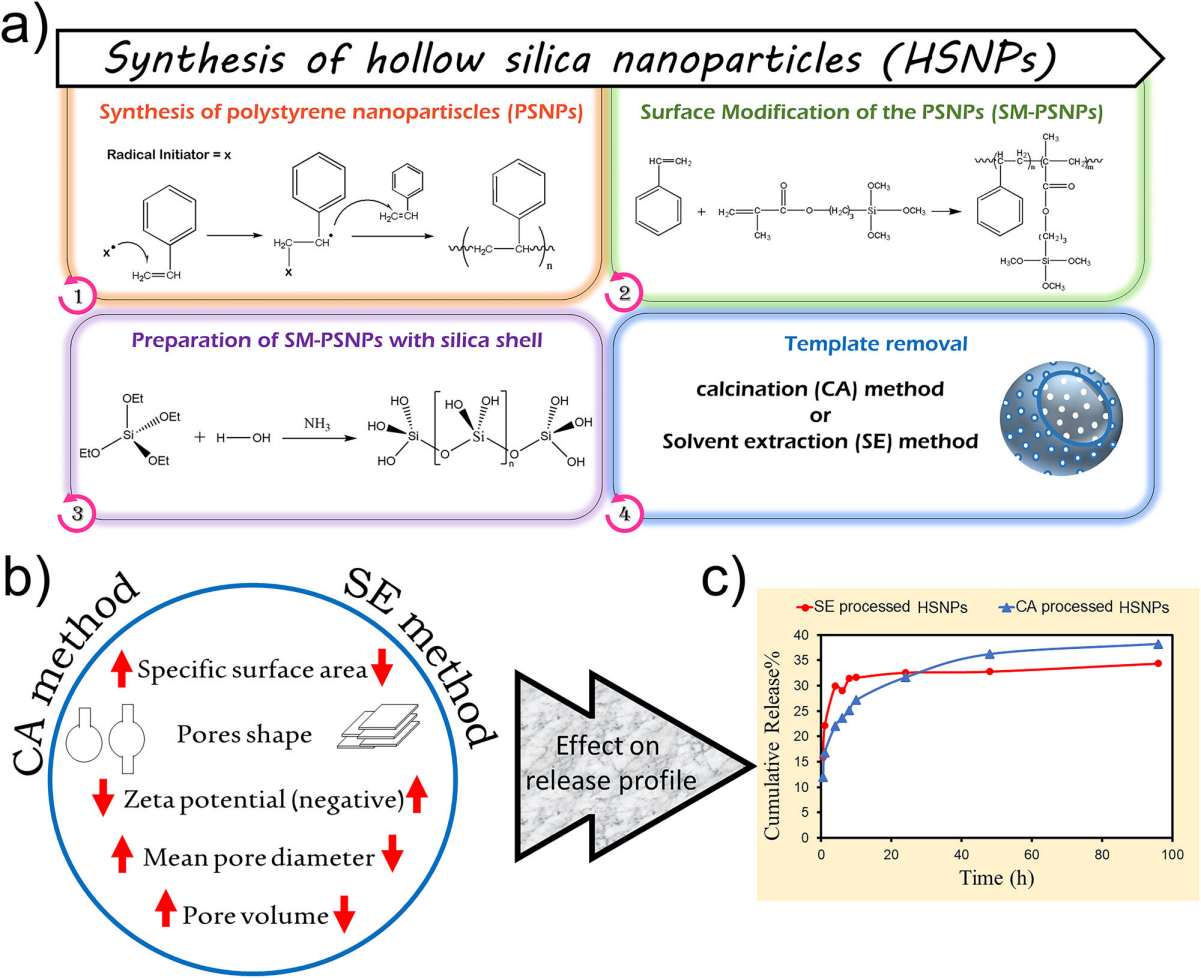
Schematic representation showing the (**a**) synthesis procedure and mechanisms for preparing HSNPs (**b**) template removal process and its impact on the characteristics of the HSNPs, and (**c**) comparison of release behaviors from nanocarriers with different structural features

The hypotheses and concepts of this study are schematically shown in Fig. 1. The synthesis of HSNPs involves four steps: the preparation of polystyrene nanoparticles (PSNPs) with a narrow size distribution as a template, silanization of the template surface, formation of a silica nanoshell, and subsequent removal of the template (Fig. 1a). The final stage plays a key role in shaping the structural features of the resulting HSNPs, including their specific surface area and pore morphology (Fig. 1b). These surface-related features significantly impact the performance of the nanoparticles in downstream applications. In particular, HSNPs synthesized with different surface and structural characteristics exhibit distinct drug release profiles, highlighting the importance of controlled template removal (Fig. 1c). These findings underscore the critical role of pore architecture and surface design in developing smart, stimuli-responsive silica-based nanocarriers.

## Materials and methods

### Materials

Styrene monomer ( ≥ 99%), sodium dodecyl sulfate (SDS, ≥85%), potassium persulfate ( ≥ 99%), 3-(trimethoxysilyl) propyl methacrylate ( ≥ 97%), and azobisisobutyronitrile (AIBN, ≥98%) were purchased from Merck Chemicals (Darmstadt, Germany) and used for synthesizing polystyrene nanoparticles (PSNPs) templates. Tetraethyl orthosilicate ( ≥ 99%), absolute ethanol ( ≥ 99.9%), and ammonia (25%) were obtained from Merck and used in the synthesis of the silica nanoshell. To extract the template, tetrahydrofuran (THF, laboratory reagent grade, >99.5%) was obtained from Fisher Scientific (Waltham, MA, USA). Doxorubicin hydrochloride (Adriabin) was procured from Sobhan Oncology Company (Tehran, Iran). All materials required for the preparation of phosphate-buffered saline, phosphate buffer, and acetate buffer solutions, including sodium chloride ( ≥ 99.5%), potassium chloride ( ≥ 99.5%), disodium hydrogen phosphate ( ≥ 99%), potassium dihydrogen phosphate ( ≥ 99%), dipotassium hydrogen phosphate ( ≥ 99%), sodium acetate ( ≥ 99%) and acetic acid (glacial, 100%), were obtained from Merck and used as received. Deionized water was obtained *via* reverse osmosis from a ZOLALAN water purification system (ZU101-B, Zolalan Sharif Plus Co., Iran).

### Synthesis of hollow mesoporous particles

Polystyrene nanoparticles (PSNPs) were synthesized as outlined in a previous study [41]. Briefly, SDS (1 g eq. to 2.95 mmol) was dissolved in deionized water (83.75 mL) and charged in a 250 mL round-bottom, 3-neck reaction flask equipped with a reflux condenser, a Teflon purge head adapter, and a thermometer. After magnetic stirring for 30 min at 400 rpm under a purge of nitrogen gas, the styrene monomer (22 mL, 190 mmol) was added, and stirring was continued for another 30 min. Then, potassium persulfate (0.1 g dissolved in 10 mL of DI water eq. to 0.37 mmol) was added dropwise within 10 min. Afterward, the reactor temperature was increased to 70 °C and maintained for 10 min while stirring. The mixture was then magnetically stirred overnight at ambient temperature to obtain a milky colloidal dispersion of PSNPS. The polymerization reaction was not terminated to facilitate the next step of silanization.

A dispersion of PSNPs (2 w/v%) in deionized water was transferred to a 250 mL 3-neck reaction flask and stirred for 40 min under a purge of nitrogen gas. Then, 10 mL of a mixture of styrene monomer, 3-(trimethoxysilyl)propyl methacrylate (80:20 volume ratio to 68.9:8.16 mole ratio), and AIBN (2.98 mmol) was added to the reaction flask and stirred for 60 min. The temperature was increased to 70 °C to initiate the polymerization reaction. Surface-modified PSNPs (SM-PSNPs) were prepared by continuing the reaction overnight. The polymerization reaction was terminated by adding methanol. The resulting SM-PSNPs were purified by washing three times with methanol and collected by centrifugation.

The SM-PSNPs were dispersed in ethanol (0.25 w/v%) via probe ultrasonication (UP400S, Hielscher Ultrasonics, Germany) for 5 min at 60% amplitude with an on/off cycle of 0.5 s. After adding ammonia (3.31, 6.63, 13.26, and 26.51 mmol of 25 wt% aqueous solution), tetraethyl orthosilicate (TEOS, 0.66, 1.11, 2.22, and 3.32 mmol dissolved in ethanol at an equal volume ratio) was subsequently added dropwise to the reaction mixture at a rate of 0.5 mL h^-1^ while magnetically stirring at 500 rpm for 24 or 48 h at ambient temperature and 40 or 60 °C until completion of the reaction. The resulting particles were washed three times with ethanol and collected by centrifugation. The effective parameters influencing the formation and structure of the silica nanoshell formed on the SM-PSNPs, including the TEOS concentration, ammonia concentration, reaction time, and temperature, were investigated. The conditions of the different runs are summarized in Table 1.

**Table 1:** Parameters investigated to process hollow nanoparticles

Run	TEOS (mM)	Ammonia (M)	Time (h)	Temperature (°C)	Template removal procedure
1	33	0.16	24	Ambient	CA (6 °C min^-1^)
2	54	0.16	24	Ambient	CA (6 °C min^-1^)
3	106	0.16	24	Ambient	CA (6 °C min^-1^)
4	155	0.16	24	Ambient	CA (6 °C min^-1^)
5	54	0.16	48	Ambient	CA (6 °C min^-1^)
6	54	0.32	24	Ambient	CA (6 °C min^-1^)
7	54	0.65	24	Ambient	CA (6 °C min^-1^)
8	54	1.29	24	Ambient	CA (6 °C min^-1^)
9	54	1.29	24	40	CA (1 °C min^-1^)
10	54	1.29	24	60	CA (1 °C min^-1^)
11	54	1.29	24	Ambient	CA (1 °C min^-1^)
12	54	1.29	24	Ambient	SE

Calcination (CA) and solvent extraction (SE) were used to prepare the HSNPs. CA was performed in a porcelain crucible within a furnace (F11L 1100, Azar Furnaces, Iran) at 550 °C for 5 h. The effect of the heating rate in the range of 1 or 6 °C min^-1^ was studied. SE was performed on dried particles (0.5 g) by adding THF (100 mL) and stirring for 24 h. The process was repeated 3 times by centrifuging and adding THF until complete template removal was achieved. The obtained nanoparticles were washed three times with ethanol. The process yield in both removal methods was calculated by measuring the weight of the samples before (*W*_*a*_) and after (*W*_*b*_) treatment according to Eq. (1).

1 $${Yeild}\left( \% \right)=\frac{{W}_{a}-{W}_{b}}{{W}_{a}}\times 100$$

### Materials characterizations

#### Fourier transform infrared spectroscopy

FTIR spectra of the samples were recorded on a Perkin Elmer Spectrum 100 device (USA) using KBr as the matrix material. The spectra ranged from 450 to 4000 cm^-1^ with a 1 cm^-1^ resolution.

#### Dynamic light scattering and zeta potential analysis

The hydrodynamic size distribution and zeta potential of the nanoparticles in DI water (25 °C) were determined *via* dynamic light scattering. An SZ-100z analyzer (Horiba Jobin Jyovin Co., France) equipped with a solid-state laser diode operating at 532 nm was used. The cumulant method was used for data analysis.

#### Electron microscopy

The morphology and size of the nanoparticles were investigated *by* field-emission scanning electron microscopy (FESEM, TESCAN, MIRA 3 LMU, Czech Republic) after gold sputtering *via* a desktop coating system (DSR1, Nanostructured Coating Company, Iran). FESEM images were taken in secondary electron (SE) mode at 15 kV and analyzed by ImageJ software (NIH, USA). More than 200 particles were analyzed to plot the size distribution histograms. For the elemental analysis of the SM-PSNPs, energy dispersive spectroscopy (EDS) was carried out after the sample was washed with methanol to remove unreacted 3-(trimethoxysilyl) propyl methacrylate. Transmission electron microscopy (TEM) was employed to study the silica nanoshell. A copper grid was used to visualize the nanoparticles under a Zeiss EM900 transmission electron microscope at 80 KeV.

#### Thermal analysis

Differential scanning calorimetry (DSC) thermograms were obtained on a Mettler DSC1/700 (Mettler Toledo, Switzerland) up to 250 °C under a nitrogen purge. The heating rate was 10 °C min^-1^. For thermogravimetric analysis (TGA), the nanoparticles were dried at 80 °C for 24 h to remove adsorbed water. Weight changes versus temperature were recorded on an SDT Q600 V20.9 Build 20 instrument (TA, USA) under purging with argon (40 mL min^-1^). The heating rate was 20 °C min^-1^ to 600 °C and the mixture was held for 10 min to stabilize the temperature, followed by air purging for another 10 min.

#### Nitrogen absorption‒desorption isotherms

Brunauer–Emmett–Teller (BET) analysis was performed on a Belsorp-Mini II instrument (Japan). The nanoparticles were degassed at 60 °C for 8 h before testing. The surface area and characteristics of the pores were determined by analyzing the nitrogen absorption‒desorption isotherms recorded with a saturation pressure of 88 kPa at 77 K.

#### X-ray diffraction analysis

XRD patterns were obtained on a Bruker D8 Advance instrument (Germany) in the 2θ range of 5–80° under Cu Kα radiation (1.5406 Å).

#### UV‒Visible spectroscopy

UV–Vis spectrophotometry was performed on a LAMBDA 950 device (LQS-ID-006, Perkin Elmer, USA). The UV–Vis spectra were analyzed to examine the removal of the PSNPs template and to quantify the drug loading percentage/release profiles. The validation of the analytical method is explained in Supporting Information S1.

### Adsorption and desorption capacity

Based on the results obtained from different experimental runs (Table 1), HSNPs prepared at a TEOS concentration of 54 mM, ammonium concentration of 1.29 M, polymerization time of 24 h, and ambient temperature (chosen for simplicity and energy efficiency owing to their minimal effect on morphology) presented the best morphology (Runs #11 and #12) and were selected for adsorption and loading capacity of a model biomacromolecules (DOX). The effects of the template removal method on nanoparticles subjected to CA (1 °C min^-1^; Run #11) or SE in THF (Run #12) were studied. For drug adsorption, 12 mg of HSNPs were immersed in 8 mL of phosphate-buffered saline containing different DOX concentrations (0.05, 0.1, 0.2, or 0.3 g L^-1^) at 37 ± 0.1 °C. Aliquots of the samples were collected from the solution at predetermined intervals, and the particles were separated *via* centrifugation. The absorbance of each sample was subsequently measured at a wavelength of 480 nm *via* validated UV–Vis spectrophotometry, and the DOX concentration in the supernatant was determined using a standard curve. (see Supplementary Information S1). The weight of unencapsulated DOX was calculated by multiplying the measured concentration in the supernatant by the volume of the solution in which the nanoparticles were immersed. The weight of loaded DOX was obtained by subtracting the unencapsulated drug from the initial weight. The encapsulation efficiency (EE) and loading efficiency (LE) were calculated at different intervals according to Eqs. (2) and (3):

2 $${EE}\left( \% \right)=\frac{{Weight\; of\; loaded\; DOX}}{{Initial\; weight\; of\; DOX}}\times 100$$

3 $${LE}\left( \% \right)=\frac{{Weight\; of\; loaded\; drug}}{{Weight\; of\; loaded\; nanoparticles}}\times 100$$

The release profile of DOX from HSNPs previously loaded with DOX at a concentration of 0.1 g L^-1^ for 24 h was investigated in phosphate-buffered saline (pH=7.4), phosphate buffer (pH=6.5), and acetate buffer (pH values of 5.5 and 4.5) was investigated. For the release experiments, 20 mg of DOX-loaded nanoparticles were dispersed in 10 mL of the respective buffer and incubated at 37 ± 0.1 °C. Samples were withdrawn at predetermined intervals and centrifuged to separate the nanoparticles. The volume was replaced with a fresh buffer to maintain a constant release volume. The cumulative release percentages were obtained by measuring the DOX concentration *via* UV-Vis spectroscopy at 480 nm and subsequent calculations.

In vitro DOX release data were evaluated with zero-order (*Q*_*t*_ = *W*_*0*_*-W*_*i*_ = *K*_*0*_ *t*), first-order (*LnQ*_*t*_*-LnQ*_*0*_ = *K*_*1*_ *t*), Hixson–Crowell (^*3*^*√W*_*0*_*-*^*3*^*√W*_*i*_ = *K*_*HC*_ *t*) and Higuchi (Q_t_=K_H_ t^0.5^) models. *Q*_*t*_ is the amount of drug released at time t, *Q*_*0*_ denotes the initial amount of dissolved drug, *W*_*i*_ represents the amount of drug remaining in the dosage form at time t, and *W*_*0*_ is the initial amount of the drug in the dosage form. *K*_*0*_, *K*_*1*_, *K*_*HC*_, and *K*_*H*_ are the rate constants for the zero-order, first-order, Hixon‒Crowell, and Higuchi equations, respectively [42].

### Safty assessments

For cytotoxicity screening, we followed an ISO‑aligned extraction method for DOX‑loaded samples and a pragmatic screening approach for unloaded silica. Specifically, for unloaded HSNPs (empty silica), the method included exposing L929 fibroblasts to the nanoparticles in the culture medium or using the extracts derived from the nanoparticles after incubation in the culture medium for 24 h. Fibroblasts (20–30×10³ cells/well) were seeded in 500 μL DMEM (Dulbecco's modified Eagle’s medium) supplemented with 10% fetal bovine serum (FBS) as the control group. Either the nanoparticles were added to three wells (n = 3), or the culture medium of three wells was replaced with 450 μL of the extracted medium and 50 μL of FBS. The plate was then incubated at 37 °C under 5% CO_2_ and 95% humidity. After 24 h, 50 μL of MTT solution (5 mg mL^-1^ in phosphate-buffered saline) was added to each well and incubated for 3 h. The culture medium was removed, and 0.5 mL of phosphate-buffered saline was added to each well to wash the cells. The solution in each well was subsequently discarded, 500 μL of DMSO was added to solubilize the formazan crystals. Absorbance of each well was recorded at 570 nm via an ELISA reader and compared with that of the control. The percentage of cell viability was calculated by Eq. (4):

4 $${\rm{Cell\; viability}}\left( \% \right)=\frac{{{OD}}_{570}{sample}}{{{OD}}_{570}{control}}$$

where the optical density (*OD*) represents the absorbance of the solution measured at 570 nm. For unloaded HSNPs (empty silica), we performed an initial pragmatic viability check: no fixed mass was deliberately imposed on the cells as a formal extract series; instead, and as a preliminary material‑focused screen, the operator applied a small, representative amount of particles directly to wells to confirm the absence of acute cytotoxicity under standard culture conditions. This direct check was intended only as an initial compatibility screen of the bare carrier rather than a full ISO extract series.

For the IC_50_ of DOX-loaded nanoparticles (HSNPs processed with CA and incubated for 24 h with 0.1g L^-1^ DOX), extracts from DOX‑loaded HSNPs were prepared according to ISO 10993 guidance by incubating the sample at a sample‑mass:extractant volume ratio of 0.1 g mL^‑1^ (primary stock extract: 100 mg mL^‑1^) in DMEM for 24 h at 37 °C under static conditions. From this stock, we prepared a series of six half‑log (factor=3.16) serial dilutions to span a wide exposure range: 100.0, 31.6, 10.0, 3.16, 1.00, and 0.316 mg mL^‑1^. Aliquots (100 μL) of each dilution were applied to L929 fibroblasts seeded in 96‑well plates (n = 5) and incubated for 24 h before MTT assay. Absorbance each well at 570 nm was measured using an ELISA reader. The percentage of viable cells was obtained according to Eq. (4). The DOX concentration in each extract was determined with a standard curve (explained in Supporting Information S1) and used to calculate the IC_50_ for released DOX.

### Statistical analysis

The size distribution of the particles was determined via SEM image analysis of SEM images using ImageJ software. The histogram and a cumulative frequency graph were plotted in Origin software to determine the mean and standard deviation (SD). The analysis of drug release data and cell viability assays was also performed using Origin software.

## Results and discussion

### Characterizations of hollow mesoporous nanoparticles

**Fig. 2:**
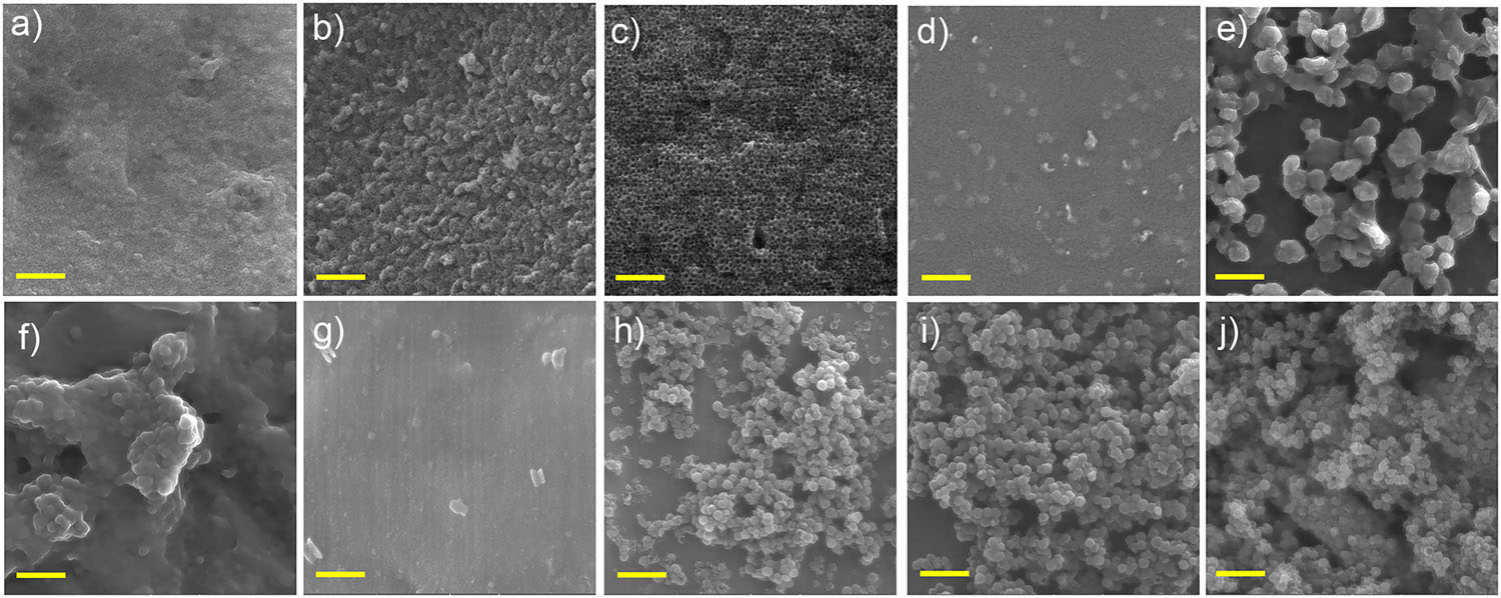
FESEM images of HSNPs prepared under different conditions (TEOS concentration, ammonia concentration, reaction time, temperature, and template removal procedure), as reported in Table 1 (Run no.): **a** #1 (33 mM, 0.16 M, 24h, Ambient, CA 6 °C min^-1^), **b** #2 (54 mM, 0.16 M, 24h, Ambient, CA 6 °C min^-1^), **c** #3 (106 mM, 0.16 M, 24h, Ambient, CA 6 °C min^-1^), **d** #4 (155 mM, 0.16 M, 24h, Ambient, CA 6 °C min^-1^), **e** #5 (33 mM, 0.16 M, 48h, Ambient, CA 6 °C min^-1^), **f** #6 (33 mM, 0.32 M, 24h, Ambient, CA 6 °C min^-1^), **g** #7 (33 mM, 0.65 M, 24h, Ambient, CA 6 °C min^-1^), **h** #8 (33 mM, 1.29 M, 24h, Ambient, CA 6 °C min^-1^), **i** #9 (33 mM, 1.29 M, 24h, 40 °C, CA 1 °C min^-1^), and **j** #10 (33 mM, 1.29 M, 24h, 60 °C, CA 1 °C min^-1^). Scale bar: 500 nm

Figure 2 shows representative FESEM images of the silica shell formed on the SM-PSNPs. The effects of different processing parameters (Table 1) were investigated. First, we investigated the influence of varying TEOS concentrations (33, 54, 106, and 155 mM) while keeping the ammonia concentration (0.16 M) and reaction time (24 hours at ambient temperature) constant. No silica shell was formed with an inadequate amount (33 mM) of TEOS (Fig. 2a). Adding more TEOS (54 mM) resulted in the formation of HSNPs (Fig. 2b); however, the particles aggregated with a broad size distribution (Fig. S4a). The observed particle aggregation can be attributed to an imbalance between rates of hydrolysis and condensation reactions. Specifically, after nucleation, hydrolysis prevails over growth and condensation, resulting in particle aggregation. At higher TEOS concentrations (106 mM), a continuous network with voids formed instead of distinct, spherical particles (Fig. 2c). Figure 2d shows that at a high TEOS concentration of 155 mM, the particle size distribution becomes non-uniform, with undesirable silica nanoparticles forming due to the preference for silica monomers produced through TEOS hydrolysis. Rather than combining with the existing silica on the surface of the template nanoparticle, these monomers tend to form new nuclei. Similarly, the reaction time affects the formation of the silica nanoshell. We found that a prolonged reaction time ( > 24 h) results in the formation of large silica particles, even those with non-spherical shapes, due to increased random collisions (Fig. 2e). The morphology of the silica nanoshell is also affected by the amount of ammonia because hydrolysis is initiated by hydroxyl anions attacking TEOS molecules, leading to a nucleophilic substitution reaction mediated by hydroxyl ions [43]. At low ammonia concentrations, the slow hydrolysis rate reduces the formation of Si–OH segments. The reduced condensation reaction occurs due to the decreased number of free silanol groups, leading to inferior silica surface coverage and particle aggregation (Fig. 2b,f,g). Nevertheless, a uniform coating is attained at a concentration of 1.29 M with no severe particle aggregation (Fig. 2h).

The effects of temperature on the size and morphology of the nanoparticles are shown in Fig. 2i, j. At elevated temperatures (40 °C and 60 °C), slightly coarser particles are formed (Fig. S4b,c) without significant influence on their morphology. On the basis of these results, we concluded that processing nanoparticles with 54 mM TEOS and 1.29 M ammonia for 24 hours at ambient temperature yields a fine coating on the template without aggregation. Therefore, these nanoparticles were selected for further studies.

**Fig. 3:**
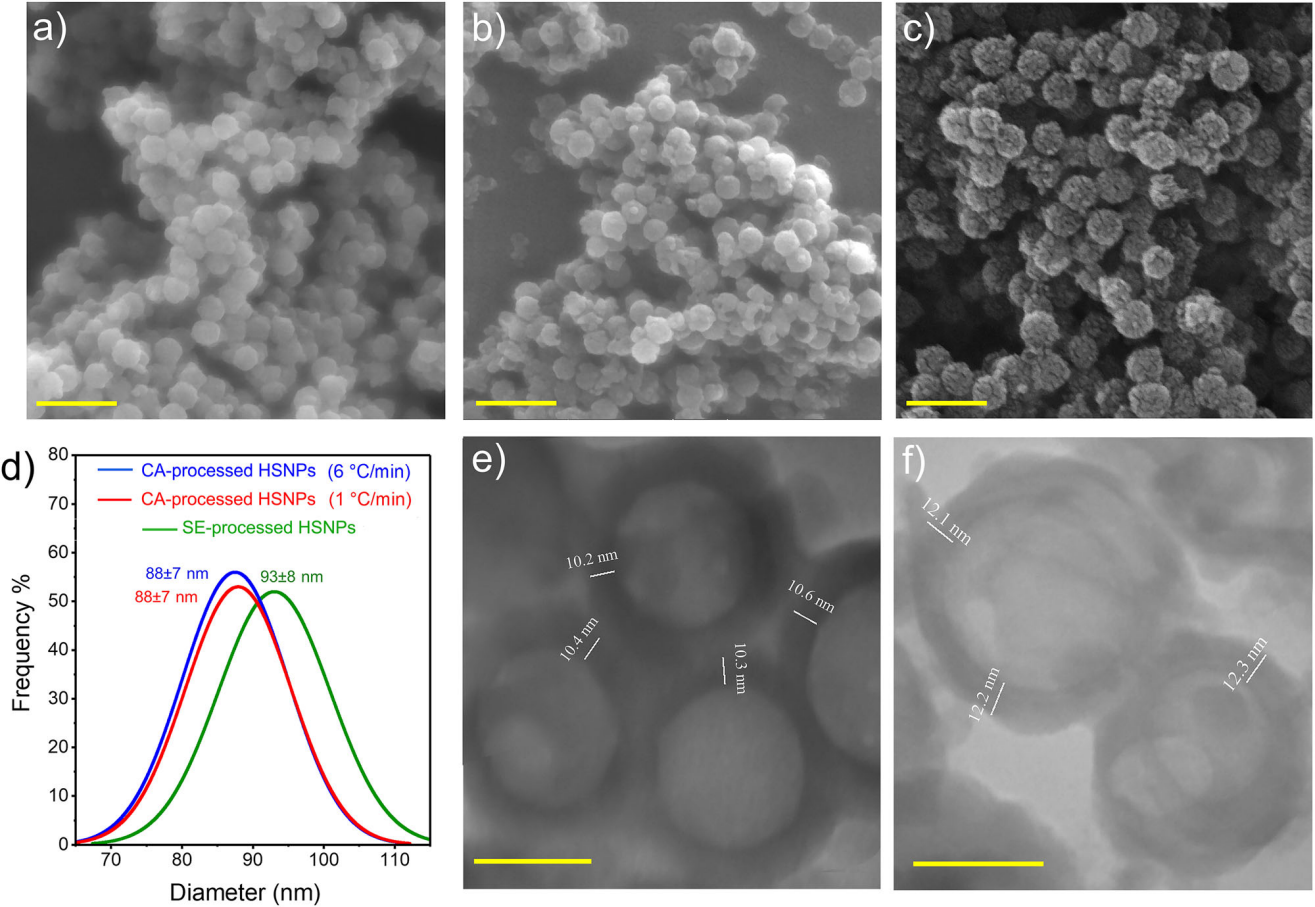
FESEM images of the HSNPs processed by (**a**) SE in THF and CA at heating rates of (**b**) 6 °C min^-1^ and (**c**) 1 °C min^-1^; scale bar: 200 nm. **d** Particle size histograms of the HSNPs. TEM images of HSNPs processed with (**e**) CA at a heating rate of 1 °C min^-1^ and (**f**) SE in THF; scale bar: 50 nm

The polymer template was removed *via* two methods: calcination (CA) and solvent extraction (SE). For the latter, the nanoparticles were dispersed in THF for various durations. We found that the complete removal of PSNPs required 96 h (Fig. S5). FESEM studies revealed spherical nanoparticles with a mean diameter of 93 nm and a relatively narrow size distribution (SD = 8) (Fig. 3a,d). Figure 3b shows CA-processed HSNPs at 550 °C at a heating rate of 6 °C min^-1^. Although spherical particles with a mean diameter of 88 nm and a narrow size distribution (SD = 7) were obtained (Fig. 3d), fracture of the silica nanoshells due to rapid polymer decomposition and buildup of the gas pressure occurred. The decomposition rate was controlled by reducing the heating rate to 1 °C min^-1^, resulting in the formation of relatively spherical nanoparticles with a mean diameter of 88 nm (SD = 7) (Fig. 3c,d). TEM studies indicated that the shell thicknesses for the SE and CA methods are approximately 12 nm and 10 nm, respectively (Fig. 3e,f). These findings were consistent with the size analysis by FESEM conducted before (Fig. S3d) and after shell coating (Fig. 3d). The estimation of the process yield *via* Eq. (1) revealed that the CA process was more efficient (49.3%) than the SE method (36.5%). The residual polymer remaining after SE processing can be attributed to covalently bound chains on the inner surface of the silica shell [41] and the high surface energy of silica [44–46], which promotes the reabsorption of extracted polystyrene chains.

**Fig. 4:**
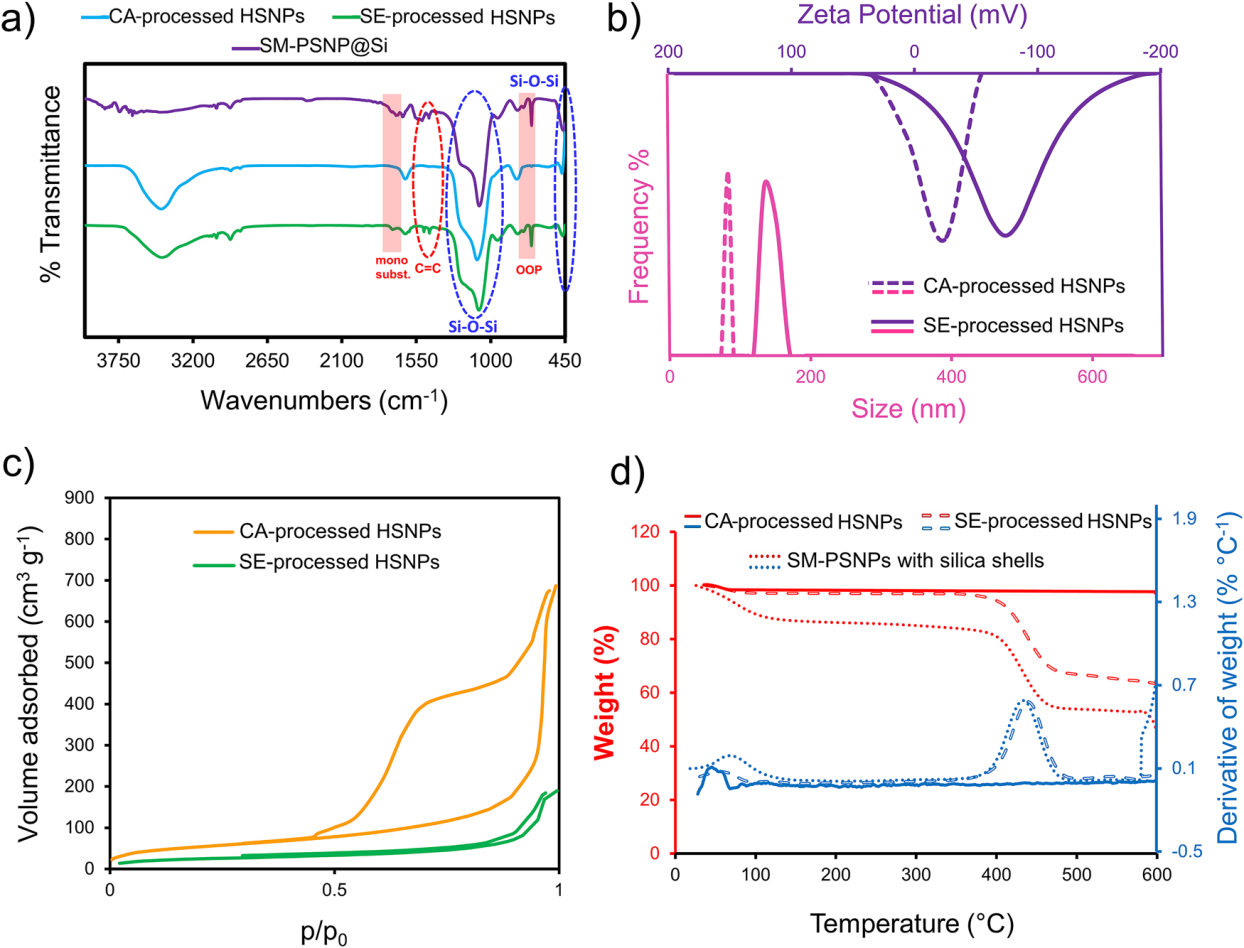
**a** FTIR spectrum of the HSNPs. OOP stands for out-of-plane vibrations of polystyrene C–H signals. **b** Particle size distribution and zeta potential of HSNPs processed by SE and CA. **c** Nitrogen absorption‒desorption isotherms and (**d**) TGA thermograms of the synthesized nanoparticles

FTIR spectroscopy (Fig. 4a) was used to determine the presence of functional groups of styrene repeating units (C6H5CH = CH2)n and SDS (CH3(CH2)11OSO3Na) on the surface of the SE-processed nanoparticles. The out-of-plane vibration bands at 698 and 757 cm^-1^, along with the bands between 1600-18000 cm^-1^, indicate the presence of a mono-substituted benzene structure, which corresponds to styrene [47]. The absorption bands at 1451 and 1492 cm^-1^ are attributed to aromatic C = C stretching [47]. The absence of these bands in the CA-processed nanoparticles reveals the complete removal of the template. Importantly, the broad bands at 1084 cm^-1^ and 470 cm^-1^ are related to the asymmetric and symmetric stretching vibrations of Si–O–Si bonds, respectively [48, 49]. The symmetric and asymmetric stretching vibrations of the sulfate group of the SDS surfactant appear at 1060 and 1215 cm^-1^, respectively. However, these bands are not observed in the spectrum because they overlap with the asymmetric vibration of Si–O–Si bands [50, 51]. Additionally, the FTIR spectrum of the silica nanoshell formed on the SM-PSNPs (SM-PSNP@Si) shows the same characteristic absorption bands observed in the SE-processed HSNPs sample. This similarity arises because the thin silica shell ( ~ 10 nm) is highly transparent to infrared radiation, allowing the vibrational modes of the underlying polystyrene core to be detected despite the coating. As a result, the aromatic C–H out-of-plane deformation bands at 698 and 757 cm^-1^ and the C = C stretching bands at 1451 and 1492 cm^-1^ are still observable in the SM-PSNP@Si spectrum. Meanwhile, the strong Si–O–Si asymmetric and symmetric stretching bands at approximately 1084 and 470 cm^-1^ remain predominant, consistent with the formation of the silica shell.

Therefore, the adsorption of the anionic surfactant (SDS) on negatively charged silica nanoparticles is possible due to hydrophobic interactions, despite electrostatic repulsion [52]. Notably, the greater zeta potential of SE-processed HSNPs (-74 mV) than CA-processed HSNPs (-14 mV) is due to the adsorption of anionic surfactants (Fig. 4b). As a result of a thicker double layer, the hydrodynamic diameter of SE processed particles was measured to be 133 ± 8 nm (microscopy image analysis revealed a diameter of 93 ± 8 nm). The nitrogen absorption-desorption isotherms of the HSNPs are shown in Fig. 4c. The type IV isotherms with a hysteresis loop characterize mesoporous materials (2-50 nm) [53]. The absence of flattening of the isotherm at high relative pressures suggests that limited adsorption and complete pore filling do not occur, presumably due to the high surface area and the presence of a macroporous structure [18]. Details about the pore shape can be inferred from the hysteresis loop. Based on the IUPAC classification [18], the hysteresis loop of SE-processed HSNPs corresponds to slit-shaped pores (H3-type). The CA-processed nanoparticles are H2-type with a steeply sloping desorption branch attributed to pore blockage in the pore mouth. Therefore, the shape of the pores is such that the size of the pore necks is much narrower than that of the main pore, i.e., the shape of the pores is ink bottle-like [54]. The average pore diameter of the nanoparticles processed with CA and SE were 20 nm and 15 nm, respectively. Therefore, the hollow silica nanoshell is mesoporous with a specific surface area and pore volume of 197.8 m^2^ g^-1^ and 1.043 cm^3^ g^-1^ (for CA-processed HSNPs) and 76.1 m2 g-1 and 0.287 cm^3^ g^-1^ (for SE-processed HSNPs), respectively. Porous HSNPs were synthesized by a surfactant-free self-templating strategy. The structure forms due to spontaneous micro-segregation in the mixture of TEOS, ethanol, and water (pre-Ouzo effect), where TEOS-rich droplets act as transient templates. Under basic conditions, hydrolysis and condensation occur at the droplet interface, producing a porous network without any surfactant or organic template [55–57]. TGA analysis of the silica-modified SM-PSNPs revealed a peak at 75 °C (Fig. 4d), which was attributed to the desorption of gases adsorbed from the air by the HSNPs. Owing to their high surface area, the porous nature of these materials facilitates the physisorption of gases from the ambient atmosphere [58]. PSNP pyrolysis occurs at approximately 430 °C, where the long chains of PSNPs are broken and converted into smaller and volatile units [59]. The complete removal of HSNPs from polystyrene chains begins at 600 °C, especially after air purging. The weight loss throughout the process was approximately 51%, which aligns with the 49.3% yield previously reported for the CA process (Section 3.2). TGA analysis of the CA-processed nanoparticles revealed only ~4% weight loss, which was partly due to adsorbed gases, with no signal of PSNP decomposition. However, in the thermogram of the SE-processed nanoparticles, the signal at approximately 430 °C indicates the presence of the residual polymer template ( ~ 34%). The discrepancy between the weight loss of SE-processed nanoparticles measured by thermogravimetry and the yield method may be due to systematic and random experimental errors.

### Adsorption capacity of nanoparticles

**Fig. 5:**
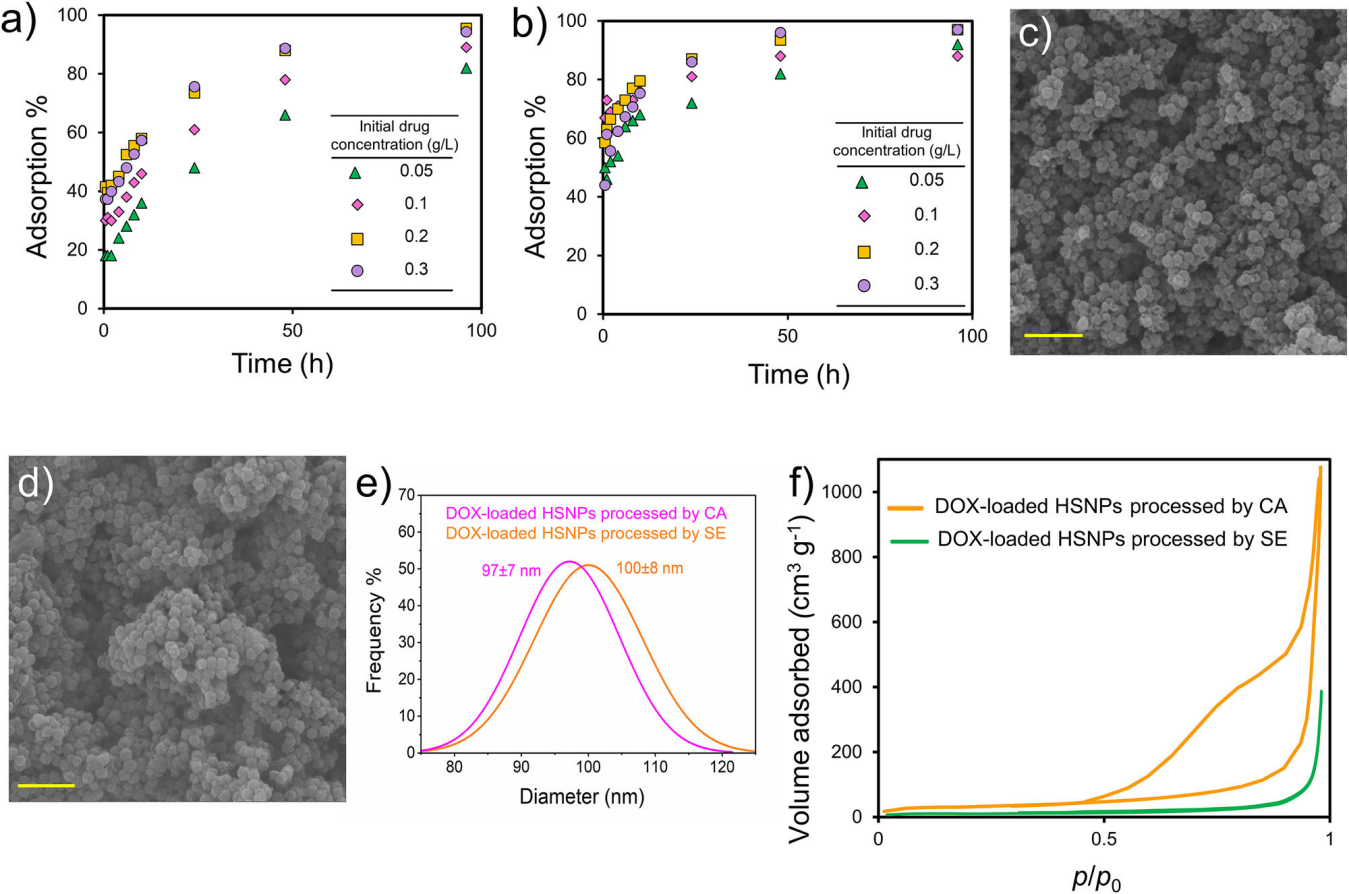
Concentration-dependent DOX adsorption on HSNPs processed by the (**a**) SE and (**b**) CA methods versus time. C: The initial concentration of DOX in solution. SEM images of (**c**) DOX-loaded HSNPs processed via the CA method and (**d**) DOX-loaded HSNPs processed via the SE method; scale bar: 500 nm. **e** Particle size distribution histograms of DOX-loaded HSNPs processed via the CA and SE methods (0.1 g L^-1^, 24 h). **f** Nitrogen adsorption-desorption isotherms of DOX-loaded HSNPs (0.1 g L^-1^, 24 h)

The nanoparticles were dispersed in a phosphate-buffered saline solution containing different DOX concentrations, and the adsorption percentages were determined over time. The results are presented in Fig. 5a, b. The encapsulation efficiency (EE) and loading efficiency (LE) of the two template removal methods are presented in Table 2.

**Table 2:** The encapsulation efficiency (EE) and loading efficiency (LE) of HSNPs at different DOX concentrations and for different durations of uptake

Time, h	Processing condition	DOX concentration, g L^-1^							
		0.05		0.1		0.2		0.3	
		Uptake, %							
		EE	LE	EE	LE	EE	LE	EE	LE
**1**	SE	18	0.6	31	2.1	39.5	5.3	37.3	7.5
	CA	46	1.5	73	4.9	63	8.4	61.3	12.3
**2**	SE	18	0.6	30	2	42	5.6	40	8
	CA	52	1.7	69	4.6	66.5	8.9	55.7	11.1
**4**	SE	24	0.8	33	2.2	45	6	43.3	8.7
	CA	54	1.8	71	4.7	70	9.3	62.3	12.5
**6**	SE	28	0.9	38	2.5	52.5	7	48	9.6
	CA	64	2.1	73	4.9	73	9.7	67.3	13.5
**8**	SE	32	1.1	43	2.9	55.5	7.4	52.7	10.5
	CA	66	2.2	73	4.9	77	10.3	70.7	14.1
**10**	SE	36	1.2	46	3.1	58	7.7	57.3	11.5
	CA	68	2.3	76	5.1	79.5	10.6	75.3	15.1
**24**	SE	48	1.6	61	4.1	73.5	9.8	75.7	15.1
	CA	72	2.4	81	5.4	87	11.6	86	17.2
**48**	SE	66	2.2	78	5.2	88	11.7	88.7	17.7
	CA	82	2.7	88	5.9	93.5	12.5	96	19.2
**96**	SE	82	2.7	89	5.9	95.5	12.7	94.3	18.9
	CA	92	3.1	88	5.9	97	12.9	97	19.4

The absorption curve showed a logarithmic increase and a DOX concentration-dependent behavior. The CA-processed nanoparticles exhibited faster adsorption and higher loading efficiency at the early stages, with less dependency on the DOX concentration. However, at prolonged durations ( ~ 96 h), the amount of DOX adsorbed was comparable to that of the SE-processed samples, particularly at higher drug concentrations. The difference in the DOX loading behavior was associated with the specific surface area and the shape and size of the pores in the silica shell. The greater specific surface area and larger pore sizes of the CA-processed HSNPs improved the uptake kinetics. The high encapsulation efficiency of DOX is mainly due to the properties of the carrier and the drug. HSNPs, with their high surface area, mesoporous structure, and two accessible surfaces, provide abundant adsorption sites, while the negatively charged silanol groups enable strong electrostatic and hydrogen bonding interactions with positively charged DOX. This effect is further enhanced by the use of a relatively high initial drug concentration and an extended incubation time.

The particle size distribution and nitrogen absorption‒desorption isotherms of the DOX-loaded nanoparticles (0.1 g L^-1^ DOX for 24 h) were studied. Figure 5c, d shows the FESEM images of the DOX-loaded HSNPs processed by CA and SE, respectively, and Fig. 5e shows their particle size distribution. The findings indicated that the average size of the DOX-loaded HSNPs was slightly greater than that of the as-synthesized nanoparticles. DOX adsorption results in a slight increase in the zeta potential (Fig. S6). A significant reduction in the specific surface area was also measured via BET (Fig. 5f and Table 3). A larger initial surface area and pore volume lead to greater DOX adsorption, resulting in a more significant reduction in the specific surface area, as observed for the CA-processed nanoparticles. Interestingly, despite the reduction in surface area, an increase in pore volume is often observed. This observation can be explained by the formation of pore-like irregularities on the surface of the drug-loaded nanoparticles. This phenomenon can be attributed to the formation of pore-like irregularities, are caused by the deposition or crystallization of DOX on the nanoparticle. Similar phenomena have been reported not only for drug loading but also in other studies following metal loading, where surface roughness and pore-like structures were observed post loading [60, 61]. Although buffer ions (Na^+^ and K^+^) could, in principle, contribute to pore volume changes through crystallization, their hydrated nature and limited interaction strength render them far less competitive than doxorubicin, which exhibits both electrostatic interactions and hydrogen bonds. Thus, the observed changes are primarily attributed to the formation of DOX crystalline structures. In other words, these irregularities or textural mesopores, contribute to a increase in pore volume without necessarily increasing the specific surface area [62]. The average pore diameter can be determined using the classical Gurvitsch method [62], as given in Eq. (5).

5 $$D=\left(\frac{4\times {\rm{V}}}{{\rm{S}}}\right)$$

where *D* is the average pore diameter, V is the pore volume, and S is the specific surface area determined from the BET method. Therefore, an increase in the apparent diameter of the mesopore structure is expected [62, 63], as shown in Table 3. Moreover, several studies have likewise reported an increase in the average pore diameter following drug loading. According to their interpretations, this effect is likely due to the complete occupation of smaller pores by the drug molecules, while larger pores remain partially unfilled. Consequently, the apparent pore diameter increases, reflecting the shift in pore size distribution rather than the creation of entirely new pores [64–66].

**Table 3:** BET pore characteristics of the HSNPs before and after DOX loading (0.1 g L^-1^, 24 h)

DOX loaded	Processing condition	Specific surface area, m^2^ g^-1^	Pore volume, cm^3^ g^-1^	Average pore diameter, nm
**As-synthesized**	CA	197.8	1.0	21.1
	SE	76.1	0.3	15.0
**DOX encapsulation**	CA	109.3	1.7	61.0
	SE	47.4	0.6	50.3

**Fig. 6:**
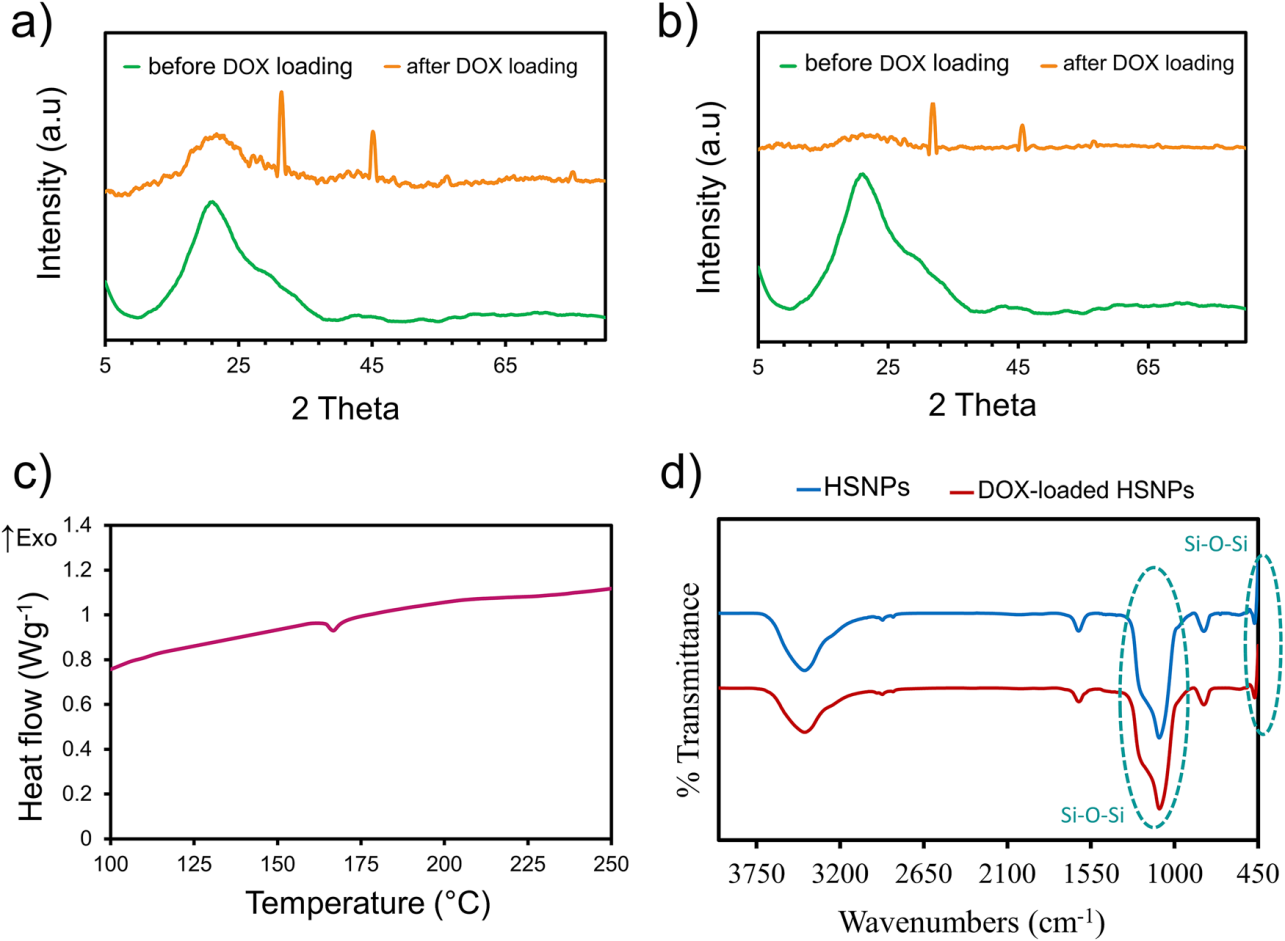
XRD patterns of HSNPs processed by (**a**) CA and (**b**) SE methods before and after DOX loading (0.1 g L^-1^, 24 h). **c** DSC curves of drug-loaded HSNPs processed by the CA method. **d** FTIR spectra of HSNPs and DOX-loaded HSNPs processed by the CA method

Figure 6a, b shows the XRD patterns of the HSNPs before and after DOX loading. Considering that the silica nanoshell is amorphous, the observation of two sharp peaks at 32° and 45° in the XRD diffractograms after drug loading reveals the presence of DOX in its crystalline form [67]. Although these peaks in the XRD pattern may be attributed to residual buffer salts, it should be noted that the crystalline endothermic event observed in the DSC thermogram (Fig. 6c) cannot originate from such salts, as their melting points occur at significantly higher temperatures. This DSC signal, therefore, reinforces the conclusion that the observed crystallinity arises from the drug itself rather than from buffer residues. The reason for the formation of drug crystals is confinement, which affects the crystallization process. Crystallization in confined volumes is an interesting process that occurs in nature [68]. Therefore, the crystallization process can occur in small volumes instead of simple models such as bulk solutions. The systems that make such a process possible are materials that are confined in at least one dimension, and the interfacial energy between the confining system and the crystalline material and the geometry in which the crystalline material is confined determine the degree of crystallization. The mass transport from the medium in the porous material until it reaches the supersaturated state resulting in growth and crystallization processes [69]. The energy barrier caused by the balance of volume-free energy and surface energy, which hinders nucleation and growth, disappears at a critical size (up to tens of nanometers in the case of organic crystal nuclei); in other words, the energy barrier compensates for the unfavorable surface energy with volume free energy [70]. The confinement growth effect can be investigated by examining the crystal properties, including the melting and freezing points. The DSC thermogram of the DOX-loaded HSNPs processed by the CA revealed a melting temperature of 167 °C (Fig. 6c), which was significantly lower than that of bulk DOX (205 °C) [71]. The decrease in the melting temperature might be attributed to the smaller drug crystal sizes (increased surface-to-volume ratio) due to the growth of the DOX crystals confined to the HSNPs [70]. FTIR studies (Fig. 6d) revealed the aliphatic C‒O stretching vibration of DOX at 1120 cm^-1^ [47]. Asymmetric absorption of C–O–C and C–N bond vibration should appear at around 1120 cm^-1^ and 1000–1350 cm^-1^, respectively [47]; however, these peaks overlap with the broad Si–O–Si band [48, 49]. A slight shift in the wavelength of the carbonyl functional group at approximately 1700 cm^-1^ due to conjugation overlaps with that at 1630 cm^-1^ [47]. The N–H stretching and bending vibrations also appear at 3500 cm^-1^ and 1580 cm^-1^, respectively [47].

### Desorption kinetics

**Fig. 7:**
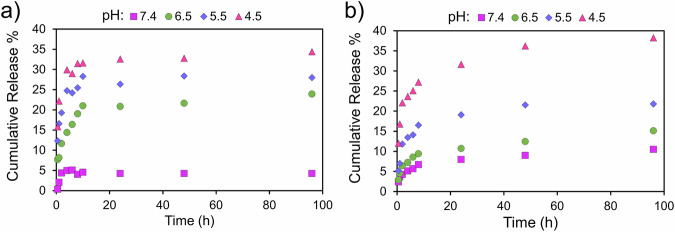
Cumulative DOX release curve from HSNPs as a function of time depending on the pH for (**a**) SE and (**b**) CA samples

The effect of pH on the desorption profile of the DOX-loaded HSNPs is shown in Fig. 7a, b. The results indicate the influence of the template removal method and pH on the release kinetics. In the buffer solution at pH 7.4, the release proceeds slowly, reaching only 10% for CA and 3% for SE after 96 hours. For SE-processed nanoparticles, 20 mg of DOX-loaded nanoparticles contained 0.82 mg of DOX, of which 0.025 mg (2.5 µg mL^-1^) was released over 96 h at pH 7.4. In comparison, 20 mg of CA-processed nanoparticles contained 1.08 mg of DOX, releasing 0.108 mg (10.8 µg mL^-1^) over the same period. Nevertheless, the kinetics are enhanced in an acidic solution (pH=4.5), approaching 40% and 37% for TE and SE, respectively. The greater burst release from the SE-processed HSNPs is attributed to the greater amount of drug adsorbed on the surface. Therefore, it can be concluded that the CA-processed HSNPs exhibit pH-responsive behavior in a more sustained way. The sustained release mechanism in acidic media is related to increased solubility in the medium, which will be discussed in Section 3.5. Since cancerous tissue is more acidic [72], this pH-dependent release helps with the targeted delivery of the drug from the HSNP carrier to the target tissue.

The desorption kinetics were studied using different models, including zero-order, first-order, Hixson–Crowell, and Higuchi equations. Table 4 presents the fitting coefficients of determination and rate constants.

**Table 4:** The coefficient of determination and rate constants obtained by fitting the release data with different kinetic models

Kinetic models			Zero-order		First-order		Hixson–Crowell		Higuchi	
		pH	R^2^	K_0_	R^2^	K_1_	R^2^	K_HC_	R^2^	K_H_
**SE processed HSNPs**	**Early stage**	**7.4**	0.45	9×10^-4^	0.40	0.226	0.45	9×10^-4^	0.61	0.004
		**6.5**	0.97	0.003	0.91	0.104	0.98	0.003	0.99	0.011
		**5.5**	0.84	0.003	0.74	0.083	0.85	0.003	0.92	0.012
		**4.5**	0.47	0.003	0.46	0.049	0.48	0.003	0.61	0.011
	**Late stage**	**7.4**	0.8	1×10^-4^	0.61	0.026	0.80	1×10^-4^	0.90	0.002
		**6.5**	0.95	1×10^-4^	0.96	0.006	0.95	1×10^-4^	0.90	0.002
		**5.5**	0.90	2×10^-4^	0.34	8×10^-4^	0.91	2×10^-4^	0.95	0.003
		**4.5**	0.99	5×10^-4^	0.97	0.009	0.99	5×10^-4^	0.99	0.007
**CA processed HSNPs**	**Early stage**	**7.4**	0.87	0.004	0.82	0.113	0.92	0.002	0.94	0.014
		**6.5**	0.89	0.006	0.78	0.13	0.90	0.003	0.96	0.023
		**5.5**	0.84	0.011	0.75	0.133	0.85	0.005	0.92	0.04
		**4.5**	0.79	0.013	0.71	0.087	0.80	0.006	0.89	0.051
	**Late stage**	**7.4**	0.88	2×10^-4^	0.81	0.008	0.86	8×10^-5^	0.96	0.003
		**6.5**	0.98	4×10^-4^	0.93	0.01	0.98	1×10^-4^	0.99	0.006
		**5.5**	0.98	7×10^-4^	0.99	0.012	0.98	3×10^-4^	0.97	0.011
		**4.5**	0.97	0.001	0.82	0.002	0.98	5×10^-4^	0.90	0.071

In the early stages, which are related to the surface adsorption of the drug, the release profiles of both types of HSNPs are well-fitted to the Higuchi model, indicating that diffusion is the release mechanism during these stages. However, at late stages, particularly under acidic conditions (i.e., pH 4.5), the desorption profiles of both types of HSNPs shift to follow the Hixson–Crowell model. This shift suggests that drug dissolution becomes the desorption mechanism at this stage. As a result, the crystalline loading of the drug significantly influences the release mechanism, leading to a transition from diffusion release in the early stages to dissolution release in acidic environments during the late stages.

### Effects of processing conditions on the structure and surface chemistry of nanoparticles

**Fig. 8:**
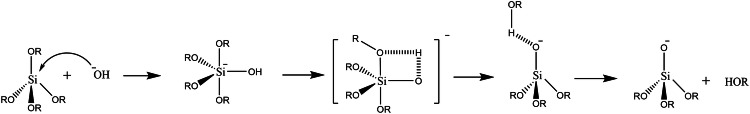
Mechanism of TEOS hydrolysis under basic conditions

A silica nanoshell was formed on the surface of polystyrene nanoparticles through the hydrolysis and condensation of alkoxysilanes under basic catalytic conditions [73]:

6 $${{\rm{NH}}}_{3}+{{\rm{H}}}_{2}{\rm{O}}\to {{\rm{NH}}}_{4}^{+}+{{\rm{OH}}}^{-}$$

7 $$\mathrm{Si}{(-\mathrm{OR})}_{4}+4\,{{\rm{H}}}_{2}{\rm{O}}\to \mathrm{Si}{(-\mathrm{OH})}_{4}+4\,{\rm{R}}-\mathrm{OH}$$

8 $${(\mathrm{OH})}_{3}\mathrm{Si}-\mathrm{OH}+{\mathrm{OH}}^{-}\to {(\mathrm{OH})}_{3}\mathrm{Si}-{{\rm{O}}}^{-}$$

9 $${(\mathrm{OH})}_{3}\mathrm{Si}-{{\rm{O}}}^{-}+\mathrm{HO}-\mathrm{Si}{(\mathrm{OH})}_{3}\to {(\mathrm{OH})}_{3}\mathrm{Si}-{\rm{O}}-\mathrm{Si}{(\mathrm{OH})}_{3}+{\mathrm{OH}}^{-}$$

10 $${(\mathrm{OH})}_{3}\mathrm{Si}-{O}^{-}+\mathrm{RO}-\mathrm{Si}{(\mathrm{OR})}_{3}\to {(\mathrm{OH})}_{3}\mathrm{Si}-O-\mathrm{Si}{(\mathrm{OR})}_{3}+{\rm{R}}-{{\rm{O}}}^{-}$$

where *R* represents alkyl (ethyl) groups. In these reactions, Eq. (6) shows the ionization of ammonia, Eq. (7) the hydrolysis of OR groups, Eq. (8) the ionization of the hydrolyzed monomer, Eq. (9) the water condensation, and Eq. (10) the alcohol condensation. These reactions usually occur simultaneously and sequentially, each with distinct kinetic characteristics. During hydrolysis and condensation reactions under alkaline conditions, nucleophilic hydroxyl groups, produced from the dissociation of water into acidic and its conjugate base forms, attack silicon atoms in alkoxysilane or silanol functional groups [73]. The proposed reaction mechanism for silane polymerization involves an S_N_2 reaction with pentavalent or hexavalent intermediates [73]. A schematic representation of this mechanism is shown in Fig. 8.

Depending on the reaction conditions, hydrolysis can lead to the protonation of all the alkoxy groups into the corresponding alcohols. As the hydrolysis reactions proceed, the concentration of the precursor [Si(OH)_4-y_(OR)_y_] rapidly increases and approaches a critical supersaturation level. After this stage, the growth process becomes dominant and continues until all of the precursors are consumed. The resulting silicic acid forms siloxane polymers *via* a condensation reaction mechanism [74]. The first hydrolysis step is rate limiting, which controls the overall polymerization kinetics [73, 75]. The reaction rate for hydrolysis and condensation depends on the concentrations of ammonia, water, and TEOS according to the following equation [76]:

11 $${Rate}=k{\left[{H}_{2}O\right]}^{\propto }{\left[{{NH}}_{3}\right]}^{\beta }{[{TEOS}]}^{\gamma }$$

[NH_3_], [H_2_O], and [TEOS] represent the concentrations of ammonia, water, and TEOS, respectively. k is the rate constant. The exponents *α*, *β*, and *γ* are the reaction orders corresponding to H_2_O, NH_3_, and TEOS, respectively. By considering the Arrhenius law [77] for the hydrolysis and condensation reactions, the rate of silane polymerization can be written as:

12 $${Rate}=A\exp \left(-\frac{{E}_{a}}{{RT}}\right){\left[{H}_{2}O\right]}^{\propto }{[{{NH}}_{3}]}^{\beta }{[{TEOS}]}^{\gamma }$$

*A*, *E*_*a*_, *R*, and *T* denote the Arrhenius factor, activation energy, universal gas constant, and absolute temperature, respectively. It has been reported that the reaction rate for TEOS is first-order, i.e., *γ* = 1 [78]. The values of *α* and *β* exponents are reported as 1.267 and 0.971 for the hydrolysis reaction and 1.196 and 0.7854 for the condensation reaction, respectively. The activation energies of the hydrolysis and condensation reactions are reported to be 25.2 and 33.2 kJ mol^-1^, with Arrhenius factors of 74.36 and 19408, respectively [73]. Therefore, the hydrolysis rate increases with increasing concentration of TEOS, resulting in the formation of free silica nanoparticles and the uncontrolled growth of the particles and shells. Similarly, increasing the ammonia concentration accelerated the hydrolysis reaction, leading to a rapid increase in the concentration of [Si(OH)_4-y_(OR)_y_]. When the concentration exceeds the supersaturation threshold, the consumption rate of the precursor species through condensation becomes relatively fast. Under these conditions, favorable homogeneous nucleation occurs, facilitating the growth of silica on SM-PSNPs. Additionally, silica nucleation is suppressed, allowing for selective deposition on the template particles. This controlled growth process ultimately reduces particle aggregation. These findings indicate that maintaining the catalyst concentration (or, equivalently, the pH) within a specific range is crucial. This ensures a balance between silica nucleation and growth, which is governed by the hydrolysis and condensation rates. Consequently, monodisperse core-shell particles with uniform shells can be obtained at an optimal ammonia concentration of 1.29 M in this investigation.

Removing the polymer template *via* the CA and SE methods resulted in HSNPs with different interfacial characteristics. The lower specific surface area and pore volume in the SE method than in the CA method can be attributed to the residual PS chains on the inner surface of the nanoparticles and the pores of the mesoporous silica shells. In other words, the readsorption of PSNPs on the surface and even the pores of the silica shell results in a reduction in the specific surface area and pore diameter/volume. The hysteresis loops of the HSNPs processed *via* the SE and CA methods are similar at a relative pressure of ~0.3. Mesoporous materials usually exhibit a nitrogen isotherm hysteresis loop (at 77 K) within a relative pressure range of 0.4 to 0.5 [18]. The adsorbent properties, including the conduit characteristics and the cavity dimensions, affect the position, shape, and size of the hysteresis loop [79]. Considering the bottle-like pore shape of CA-processed HSNPs, the relative pressure is affected by the neck dimensions (width and length) through cavitation or pore-blocking mechanisms [80]. The former mechanism occurs at a relative pressure of 0.4 [79], whereas the latter is observed in bottle-like pores with neck sizes smaller than a critical size (3–4 nm), exhibiting a knee-shaped hysteresis loop with reduced desorption pressure [80]. The H2-type hysteresis loop in the CA-processed HSNPs confirmed the cavitation-like pore-blocking mechanism. In agreement with previous studies [81], in the case of slit-shaped pores (SE method), the length and width of the pores affect the isotherm. The longer the pores are, the larger the hysteresis loop becomes, and as the pore width of the hysteresis loop decreases, it shifts to a lower relative pressure. As a result, HSNPs processed by SE have slit-shaped pores that are small in length and width.

### Effect of pore structure and surface charge on the adsorption-desorption behavior

The surface charge of the HSNPs shows a slight increase after adsorption DOX from -14 to -3 mV for CA-processed and from -74 to -65 mV for SE-processed nanoparticles (Fig. 4b and Figure S6). Since the DOX has a positive charge, loading the drug onto HSNPs increases the charge of HSNPs. These zeta potential measurements were performed at a loading time of 24 h and an initial DOX concentration of 0.1 g L^-1^. Based on the loading and encapsulation efficiencies reported in Table 2, increasing the loading time or the initial DOX concentration would further enhance drug loading, and more pronounced changes in zeta potential can therefore be expected. Therefore, the magnitude of the changes in the surface charge of HSNPs depends on drug loading. We found that irrespective of the surface charge, both CA- and SE-processed HSNPs had favorable stability in aqueous environments owing to the thermal motion phenomenon of colloidal particles. Studies of the drug release profile indicated a burst release followed by a sustained release dependent on the template removal procedure and pH. The first stage corresponded to the release of drugs adsorbed on the nanoparticle surfaces [82]. The second stage can be attributed to the release of crystallized macromolecules within the porous structure through the dissolution mechanism, as the release profile is highly pH-sensitive (Fig. 7a, b). These findings highlight the role of pore structure in controlling drug release. Previous studies have shown that tuning the size of the pores can regulate the release rate, with larger pores in silica-based carriers generally leading to increased drug release [17, 83, 84]. In contrast, the present study reveals a different mechanism governing drug release, originating from the confinement of drug molecules within the pores. This confined environment promotes drug crystallization, which fundamentally alters the release behavior. Pore architecture plays an important role in controlling the pH-responsive behavior of the system. smaller or more tortuous pores enhance confinement effects, leading to the formation of smaller, irregular crystals with high surface-to-volume ratios. The dissolution behavior of these confined crystals with high surface-to-volume ratios is highly sensitive to the surrounding environment, causing the drug release to be strongly responsive to pH. DOX is a weak base with a pK_a_ of 8.82 in N/20 NaOH solution [71, 85]. According to the Henderson–Hasselbalch equation [86, 87], weakly basic drugs are more soluble at pH < pK_a_.

13 $${pH}={{pK}}_{a}+\log \frac{[B]}{{[{BH}}^{+}]}$$

[B] and [BH + ] are the molar concentration of the undissociated base and the molar concentration of the soluble conjugate acid cation, respectively. Therefore, the dissolution of DOX with amine functional groups should be enhanced under acidic conditions. Our results support the hypothesis that a pH-sensitive nanocarrier can be achieved without secondary modification efforts by tuning the physicochemical characteristics of the nanocarrier.

### Safety concerns of hollow nanoparticles

**Fig. 9:**
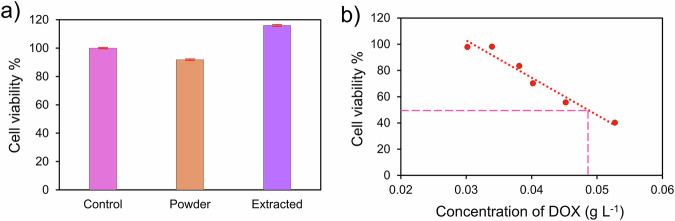
**a** L929 fibroblast viability after 24 h of culture with HSNPs or their extracts. **b** Cell viability as a function of the released DOX concentration

The biological experiments presented here were designed to answer the materials‑science questions central to this manuscript (carrier cytocompatibility and whether engineered pore/surface features produce pH‑sensitive release that can reach biologically relevant drug levels). According to ISO 10993-5, the general requirements for cell models emphasize that the cell line selected for cytotoxicity evaluation must provide reproducible and accurate responses. In the MTT cytotoxicity test section, the experimental procedure described in the standard reflects the commonly adopted practice of performing this assay with L929 cell lines in cytotoxicity evaluations. Following these criteria, we employed human dermal fibroblasts, which are widely used in MTT-based cytotoxicity assessments due to an established history in biocompatibility testing [88–90]. While our study focused on fibroblasts as a primary in vitro model, we note that evaluating additional cell types could further enhance the comprehensiveness of the biocompatibility assessment. For the unloaded HSNPs carrier, in addition to the extraction method, a small quantity of particles was applied directly to the cells to confirm the absence of acute cytotoxicity; no formal, fixed mass series was applied for the empty carrier in this exploratory screen. The empty‑carrier check returned >92% viability after 24 h (Fig. 9a), supporting the conclusion that the silica scaffold itself does not induce acute cytotoxicity under these screening conditions. For DOX‑loaded HSNPs, we used ISO 10993‑style extracts (stock extract 0.1 g mL^‑1^ with six half‑log serial dilutions: 100.0, 31.6, 10.0, 3.16, 1.00, and 0.316 mg mL^‑1^) and determined the IC50 of the released DOX using validated quantitation. These controlled extracts allow direct comparison between release kinetics and the drug concentration required to induce a biological effect. To determine the IC_50_ (the concentration that results in 50% cell viability), linear regression analysis (R^2^ > 0.96) was used. The half-maximal inhibitory concentration was attained at a DOX concentration of ~0.05 g L^-1^ (Fig. 9b). In vitro, release data indicated that this amount of drug release (per microgram of CA-processed nanoparticles incubated with 0.1 g L^-1^ DOX for 24 h) could not be achieved at pH 7.4 even after prolonged incubation (i.e., 96 hours, Fig. 7b). However, when the pH was decreased to 6.5, 5.5, and 4.5, this amount of drug was attained at 39, 4, and 0.5 hours, respectively. These results demonstrate the good biocompatibility and pH-responsive behavior of HSNPs, suggesting their potential for safe and effective drug delivery. The high cell viability ( > 92%) indicates low toxicity, supporting their suitability for biomedical applications. The controlled drug release in acidic conditions mimics tumor microenvironments, highlighting their possible therapeutic relevance. We emphasize that quantitative extrapolation from these in vitro mass concentrations to in vivo particle numbers and local tissue doses requires biodistribution, clearance, and pharmacokinetic data, which are beyond the current materials‑focused study. Addressing this gap will require a dedicated follow-up study that includes extensive cytotoxicity across multiple cell types (cancer and related healthy cells), cellular uptake and subcellular localization (confocal microscopy and TEM), hematopoietic and complement activation assays, serum protein corona characterization, and preliminary in vivo pharmacokinetics and biodistribution in a small animal model, such that these data allow for accurate in vivo dose estimation in the case of targeted drug delivery.

## Conclusions

Mesoporous hollow silica nanoparticles (HSNPs) with an average diameter of 64 ± 11 nm and a shell thickness of 10–12 nm were successfully synthesized using a polymer-template method. Spherical HSNPs with a narrow size distribution and minimal aggregation were achieved at ambient temperature by optimizing the concentrations of tetraethyl orthosilicate (TEOS) and ammonia to 54 mM and 1.29 M, respectively. Two template removal strategies, calcination (CA) and solvent extraction (SE), were evaluated. The calcination method produced HSNPs with a higher specific surface area ( > 195 m^2^ g^-1^), a larger average pore diameter ( ~ 20 nm), and an ink-bottle-like mesoporous structure. These structural features significantly influenced the adsorption and desorption behavior of the nanoparticles. Without any surface functionalization, the synthesized HSNPs exhibited pH-responsive desorption kinetics, with drug release under an acidic condition (pH=4.5) proceeding approximately four times faster than at pH=7.4. Our results further demonstrated that the hollow silica nanoparticles are safe and cytocompatibility. These findings underscore the importance of structural and surface engineering, particularly through controlled template removal, in developing smart, pH-sensitive nanocarriers without the need for chemical surface modification. While the present study demonstrates cytocompatibility and release characteristics of the carriers in vitro, comprehensive biological evaluation, including multi-cell line cytotoxicity, cellular uptake, hemocompatibility, and in vivo pharmacokinetics and biodistribution, is necessary before translation to preclinical models.

## Supplementary information

Supplementary information

## Data Availability

Data will be made available on request.
