# Supplementary material for: Template-assisted synthesis of pH-responsive hollow mesoporous silica nanocarriers: the role of engineered pores and surface characteristics
Source: J Mater Sci Mater Med. 2026 Jan 20;37(1):28. doi: 10.1007/s10856-025-06995-z (PMC12852280; doi:10.1007/s10856-025-06995-z)
Supplement: Supplementary file 1 — Supplementary information [file 10856_2025_6995_MOESM1_ESM.docx]

*Supporting information*

**Template-Assisted Synthesis of pH-responsive Hollow Mesoporous Silica Nanocarriers: The Role of Engineered Pores and Surface Characteristics**

Sahar Gooneh-Farahani^a^, Mohammad Imani^a,b^ ⃰, Morteza Daliri Joupari^c^, Abdolreza Simchi^a,d,e*^^[[1]](#footnote-1)^

^a^ Center for Nanoscience and Nanotechnology, Institute for Convergence Science & Technology, Sharif University of Technology, 14588 89694 Tehran, Iran

^b^ Novel Drug Delivery Systems Dept., Faculty of Science, Iran Polymer and Petrochemical Institute, 14977 13115 Tehran, Iran

^c^ Animal and Marine Biotechnology Dept., National Institute of Genetic Engineering and Biotechnology, 14977 16316 Tehran, Iran.

^d^ Department of Materials Science and Engineering, Sharif University of Technology, 14588 89694 Tehran, Iran

^e^ Center for Bioscience and Biotechnology, Institute for Convergence Science & Technology, Sharif University of Technology, 14588 89694 Tehran, Iran

*Corresponding Authors: [mohammad.imani@sharif.edu](mailto:mohammad.imani@sharif.edu) (M. Imani) and [simchi@sharif.edu](mailto:simchi@sharif.edu)

(A. Simchi)

**S1. Analytical method validation**

The UV‒Vis method for DOX quantitation was validated according to the ICH guidelines [1]. DOX solutions (0.004, 0.006, 0.008, 0.01, 0.02, 0.03, 0.04, 0.05, and 0.06 g L^-1^) in phosphate-buffered saline were prepared by arithmetic serial dilution of a stock solution of 2 mg mL^-1^ to construct a standard curve. The DOX solutions were prepared in five repetitions on three different days (three times on one day and two on the other two consecutive days). UV‒Vis spectra were recorded three times for each standard solution sample at wavelengths ranging from 400–800 nm. Standard curves were constructed based on the sample absorbance at λ_max_=480 nm. The limit of detection (LOD) and the limit of quantification (LOQ) were calculated from the y-intercept (*SD*) and the slope (*S)* of the five standard curves according to **Equations (S1)** and **(S2)**, respectively.

$\boldsymbol{LOD:3.3\times}\frac{\boldsymbol{SD}}{\boldsymbol{S}}$ **Eq. (S1)**

$\boldsymbol{LOQ:10\times}\frac{\boldsymbol{SD}}{\boldsymbol{S}}$ **Eq. (S2)**

The final standard curve was obtained by averaging the absorption values from five repetitions of the standard curves. All these steps were repeated by preparing standard solutions in phosphate (pH 6.5) and acetate (pH 5.5 and 4.5) buffer solutions, and for each buffer, the standard curve, LOD, and LOQ were calculated. The obtained ​​LODs and LOQs for the DOX drug in all four-buffer media are listed in **Table S1**.

**Table S1**. LOD and LOQ values for the drug DOX in phosphate-buffered saline, phosphate buffer (pH 6.5), and acetate buffer (pH 5.5 and 4.5).

|  | **Phosphate-buffered saline (pH 7.4)** | **Phosphate buffer (pH 6.5)** | **Acetate buffer (pH 5.5)** | **Acetate buffer (pH 4.5)** |
| --- | --- | --- | --- | --- |
| **LOD (g L^-1^)** | 0.00148 | 0.00088 | 0.00114 | 0.00135 |
| **LOQ (g L^-1^)** | 0.00449 | 0.00267 | 0.00345 | 0.0041 |

The same procedure was repeated to validate DOX determination (six half-log dilution samples) in culture media during in vitro studies. Briefly, DOX solutions (0.04, 0. 06, 0.08, 0.1, 0. 2, 0. 4, 0.6, and 0.8 μg μL^-1^) were prepared in DMEM by serial dilution (*n*=3). The OD of each standard solution was read at 480 nm using an ELISA reader, and a standard curve was constructed (**Figure S1)**. The amount of drug in 6 unknown samples was subsequently obtained according to the standard addition method to eliminate the matrix effect and increase the accuracy of the measurement with the help of the standard curve.


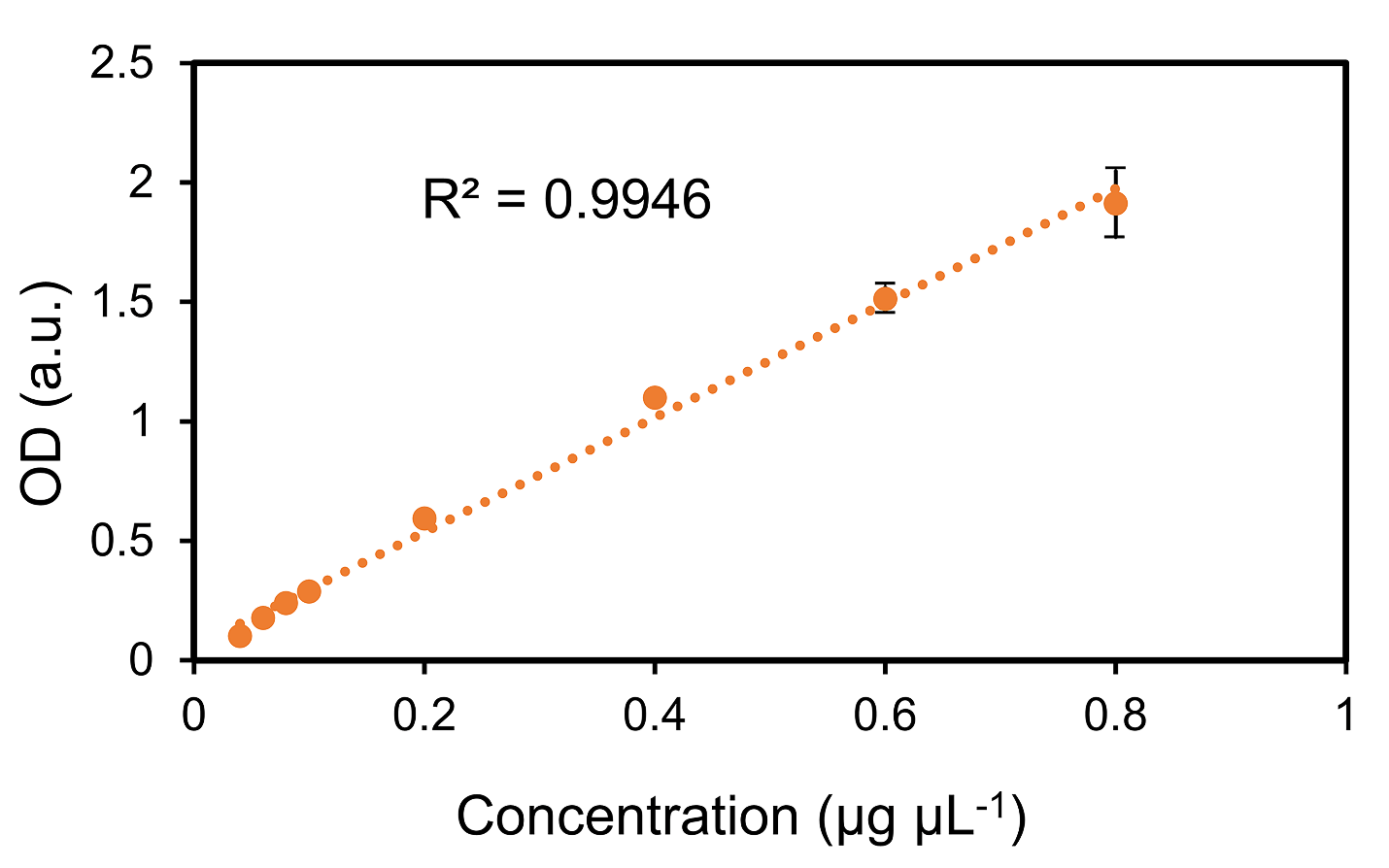


**Figure S1.** DOX standard curve in DMEM

**S2. Characterizations of PSNPs**

PSNPs were prepared through a free-radical, emulsion polymerization reaction mechanism [2], which is among the most efficient methods for producing polymer colloids. Sodium dodecyl sulfate and potassium persulfate were used as the surfactant and free radical initiator, respectively. SEM studies show that spherical PSNPs with narrow size distribution were synthesized via free radical polymerization (**Figure S2a**). Dynamic light scattering determines a mean particle size of 64 ± 11 nm (**Figure S2b**). The high zeta potential of the nanoparticles (-84 mV), which is linked to the adsorbed anionic SDS on the surface of the particles, assures the stability of the colloidal system for further processing.


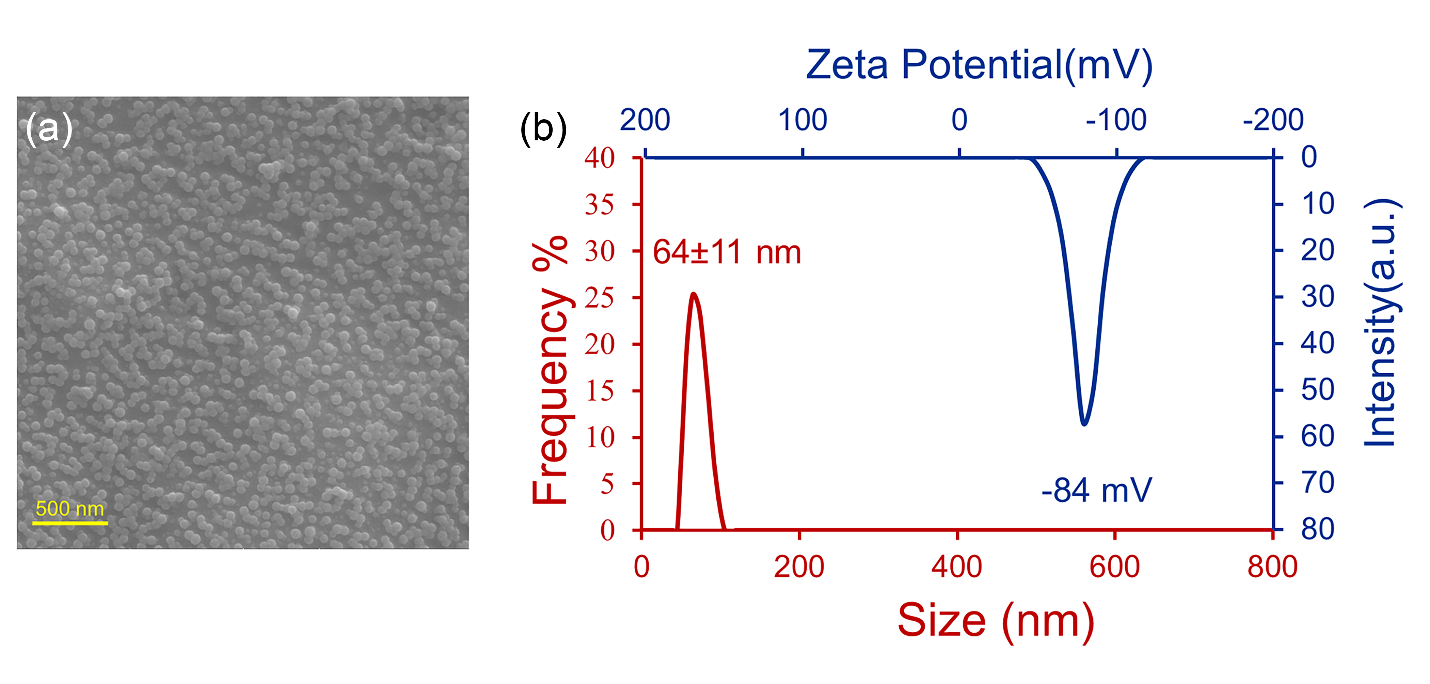


**Figure S2. (a)** A representative FESEM micrograph and **(b)** particle size distribution and zeta potential of the PSNPs.

**S3. Characterizations of SM-PSNPs**

The surface of polystyrene nanoparticles was functionalized with styrene and 3-(trimethoxysilyl)propyl methacrylate copolymer to facilitate the formation of the silica shell in the next stages. EDS affirms the presence of silicon on the surface of SM-PSNPs after washing with methanol several times (**Figure S3a**)**. Figure S3b** shows the FTIR spectrum of PS nanoparticles before and after surface modification. Two distinct bands are evident in the FTIR spectrum of the SM-PSNPs compared with those of the PSNPs. Specifically, there is a band at 1112 cm^-1^, associated with the Si–O stretching vibrations [3], and a strong band at 1726 cm^-1^, which corresponds to the carbonyl stretching vibrations[4] present in the structure of 3-(trimethoxysilyl)propyl methacrylate. The existence of these bands in the FTIR spectrum and the observation of the Si peak in the EDS analysis provide evidence for the successful surface modification of PSNPs with the 3-(trimethoxysilyl)propyl methacrylate as a silane coupling agent.

Given that SM-PSNPs serve as templates for the preparation of hollow silica particles, uniformity in particle morphology is crucial. **Figure S3c** shows the spherical morphology of SM-PSNPs. Determination of the particle size distribution ImageJ software (Ver. 1.53t, National Institutes of Health, USA) determines an average diameter of 68 ± 6 nm (**Figure S3d**). Therefore, the thickness of the silica shell is estimated to be about 4 nm.


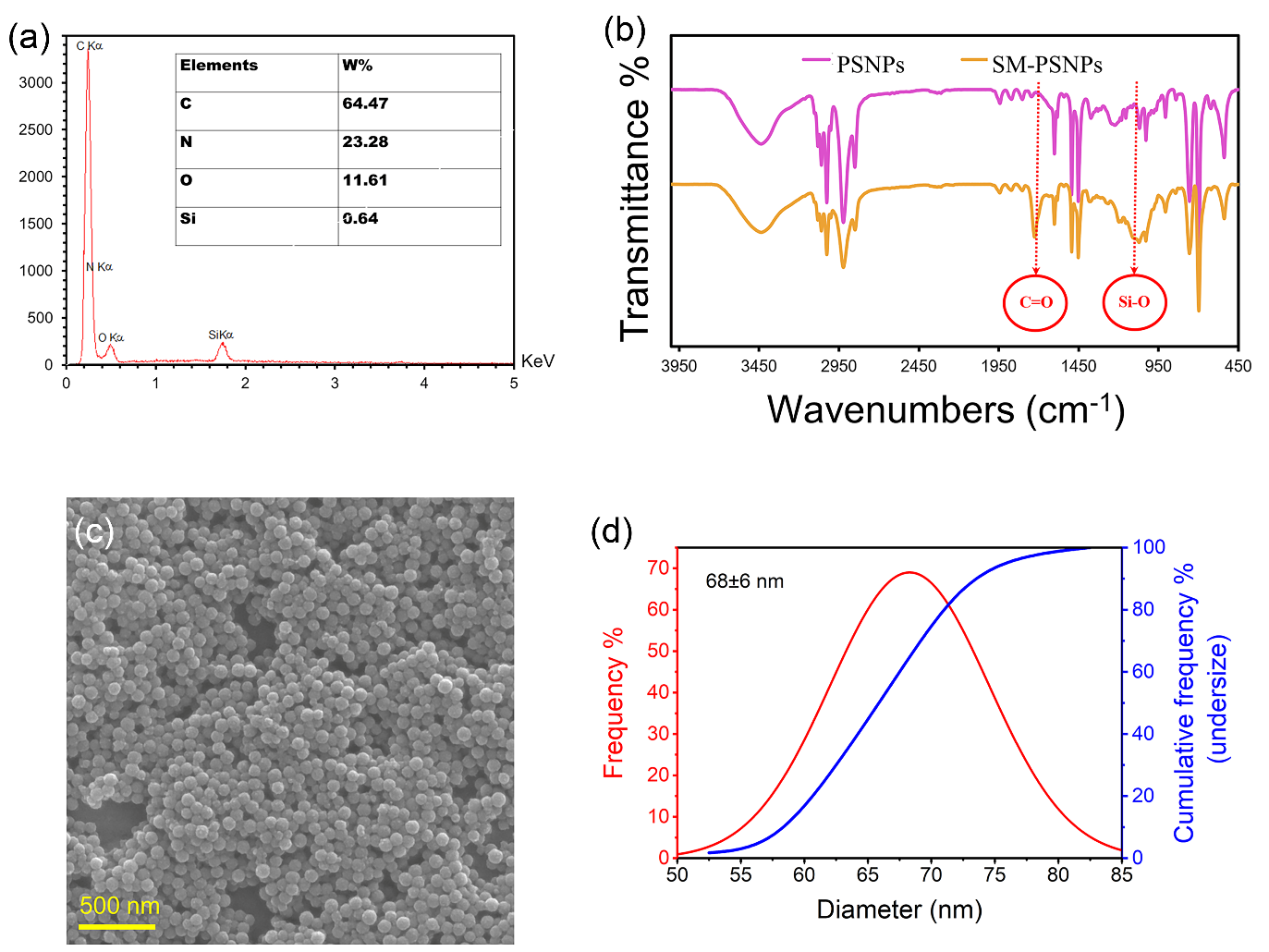


**Figure S3.** **(a)** EDS analysis, **(b)** FTIR spectra, **(c)** representative FESEM images, and **(d)** particle size distribution of the SM-PSNPs.

**S4. Characterization of HSNPs**

The particle size distribution of selected HSNPs prepared at different conditions is shown in **Figure S4**. The effect of template removal by the SE method is shown in **Figure S5**. The peak positioned at 289 nm belongs to the styrene repeating units present in the template backbone [5]. After 24 h extraction, the solvent was separated by centrifugation, analyzed, and the suspension refreshed for longer examinations. Three replicate experiments were conducted in each test to ensure repeatability. The results indicate progressive solvent extraction of PS, in which the characteristic peak is weakened until the third extraction series (**Figure S5d**) at which the peak becomes unnoticeable. The zeta potential of HSNPs after DOX loading is shown in **Figure S6**. The reduced surface charge indicates electrostatic interactions and adsorption of the macromolecules on the surface of HSNPs.


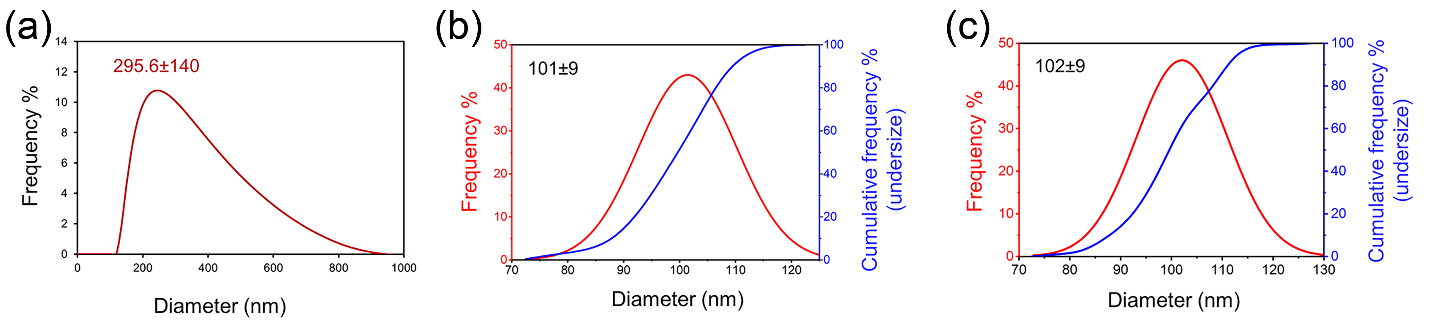


**Figure S4.** Particle size distribution of HSNPs prepared by **(a)** Run #2 (according to DLS), **(b)** Run #9 (according to image analysis), and **(c)** Run #10 (according to image analysis).


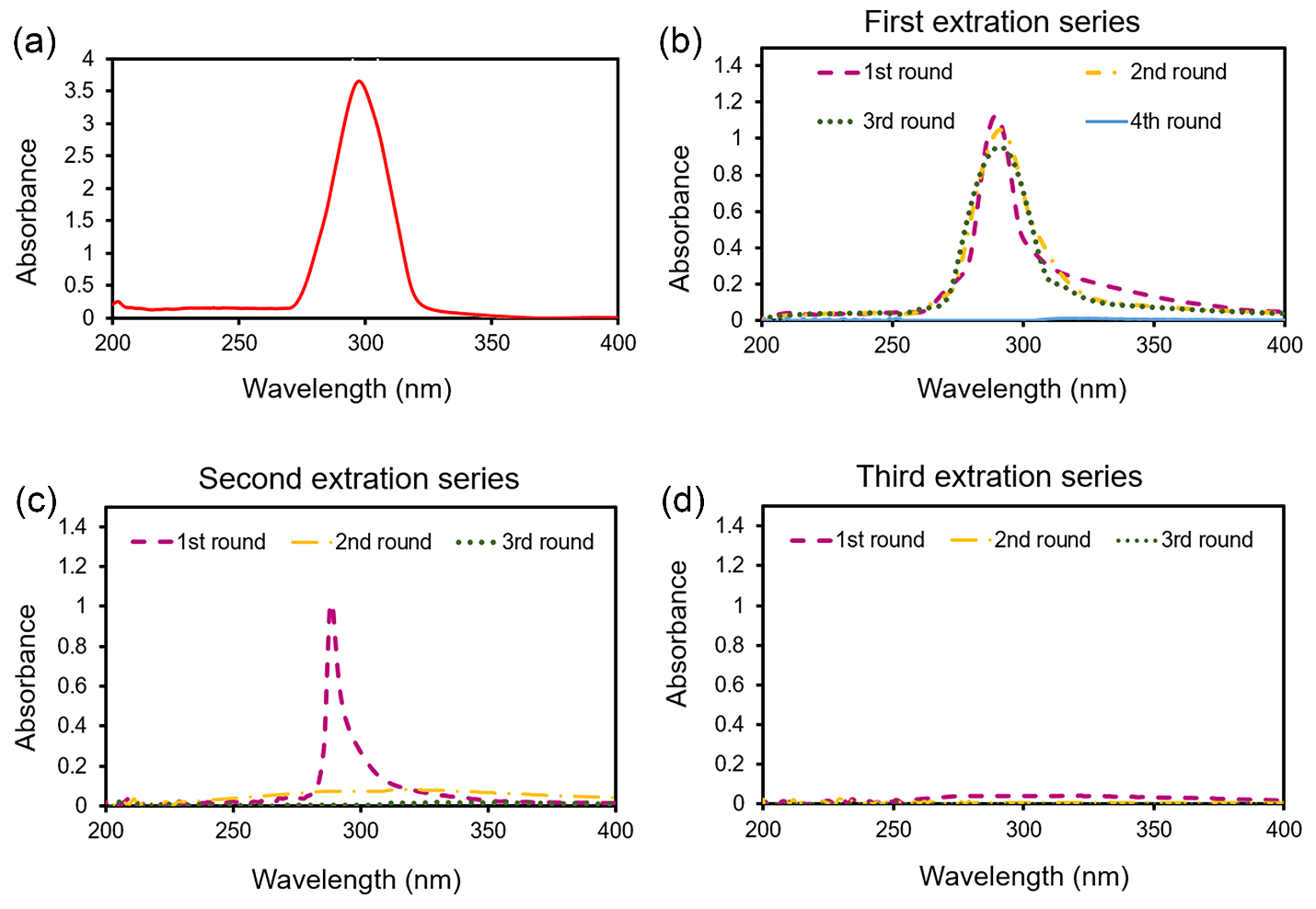


**Figure S5.** UV spectrum of SM-PSNPs in THF: **(a)** as-synthesized; **(b)** solvent extracted for 24 h, **(c)** solvent extracted for 48 h; **(d)** solvent extracted for 96 h.


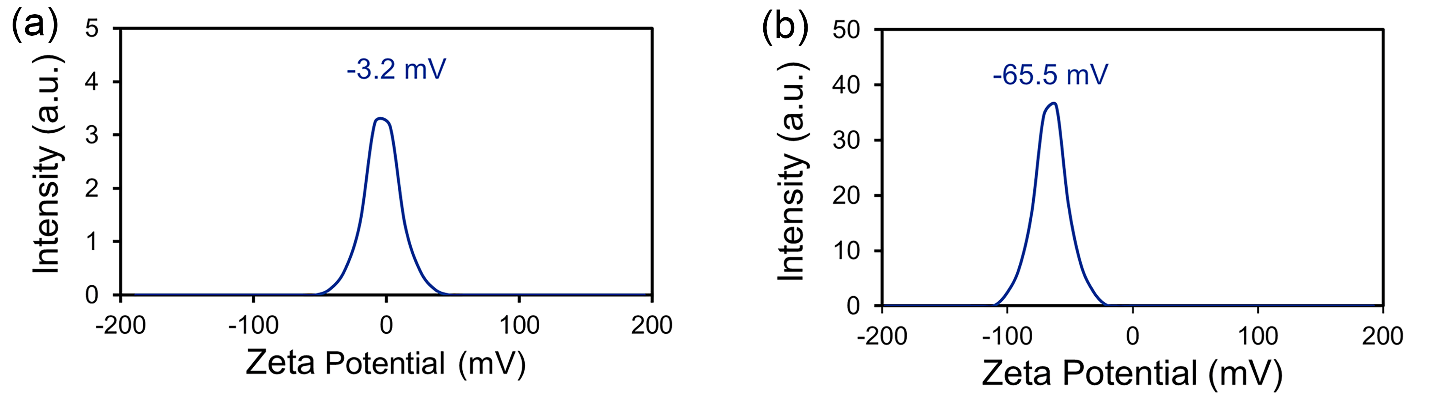


**Figure S6.** Zeta potential of DOX-loaded HSNPs processed by the **(a)** CA and **(b)** SE methods.

**References**

[1] I.H.T. Guideline, Validation of analytical procedures: text and methodology, Q2 (R1) 1(20) (2005) 05.

[2] F. Guignard, M. Lattuada, Template-assisted synthesis of Janus silica nanobowls, Langmuir 31(16) (2015) 4635-4643.

[3] A. Silva, C. Queiroz, S. Agathopoulos, R. Correia, M. Fernandes, J. Oliveira, Structure of SiO2–MgO–Na2O glasses by FTIR, Raman and 29Si MAS NMR, Journal of Molecular Structure 986(1-3) (2011) 16-21.

[4] D.L. Pavia, G.M. Lampman, G.S. Kriz, J.A. Vyvyan, Introduction to spectroscopy, Cengage learning2014.

[5] T. Li, C. Zhou, M. Jiang, UV absorption spectra of polystyrene, Polymer Bulletin 25 (1991) 211-216.

1. Current affiliation: Fraunhofer Institute for Manufacturing Technology and Advanced Materials (IFAM), 28359 Bremen, Germany; [abdolreza.simchi@ifam.fraunhofer.de](mailto:abdolreza.simchi@ifam.fraunhofer.de) [↑](#footnote-ref-1)
